# Supplementary figures and images for: Modeling microbial cross-feeding at intermediate scale portrays community dynamics and species coexistence
Source: PLoS Comput Biol. 2020 Aug 18;16(8):e1008135. doi: 10.1371/journal.pcbi.1008135 (PMC7480867; doi:10.1371/journal.pcbi.1008135)

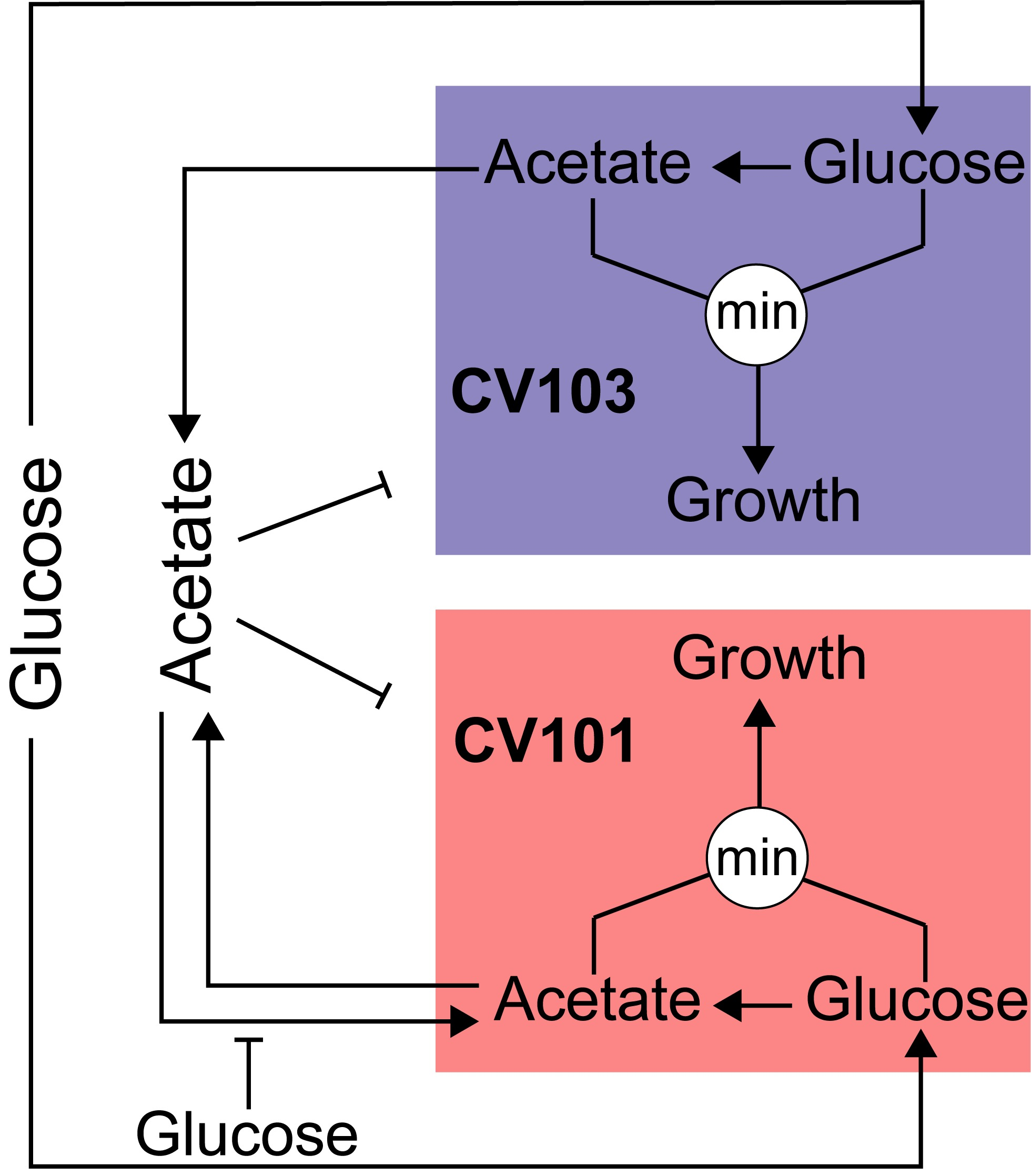

Supplement: S1 Fig — For reference, a simplified version is shown in Fig 2A of the main text. (TIF) [file pcbi.1008135.s001.tif]

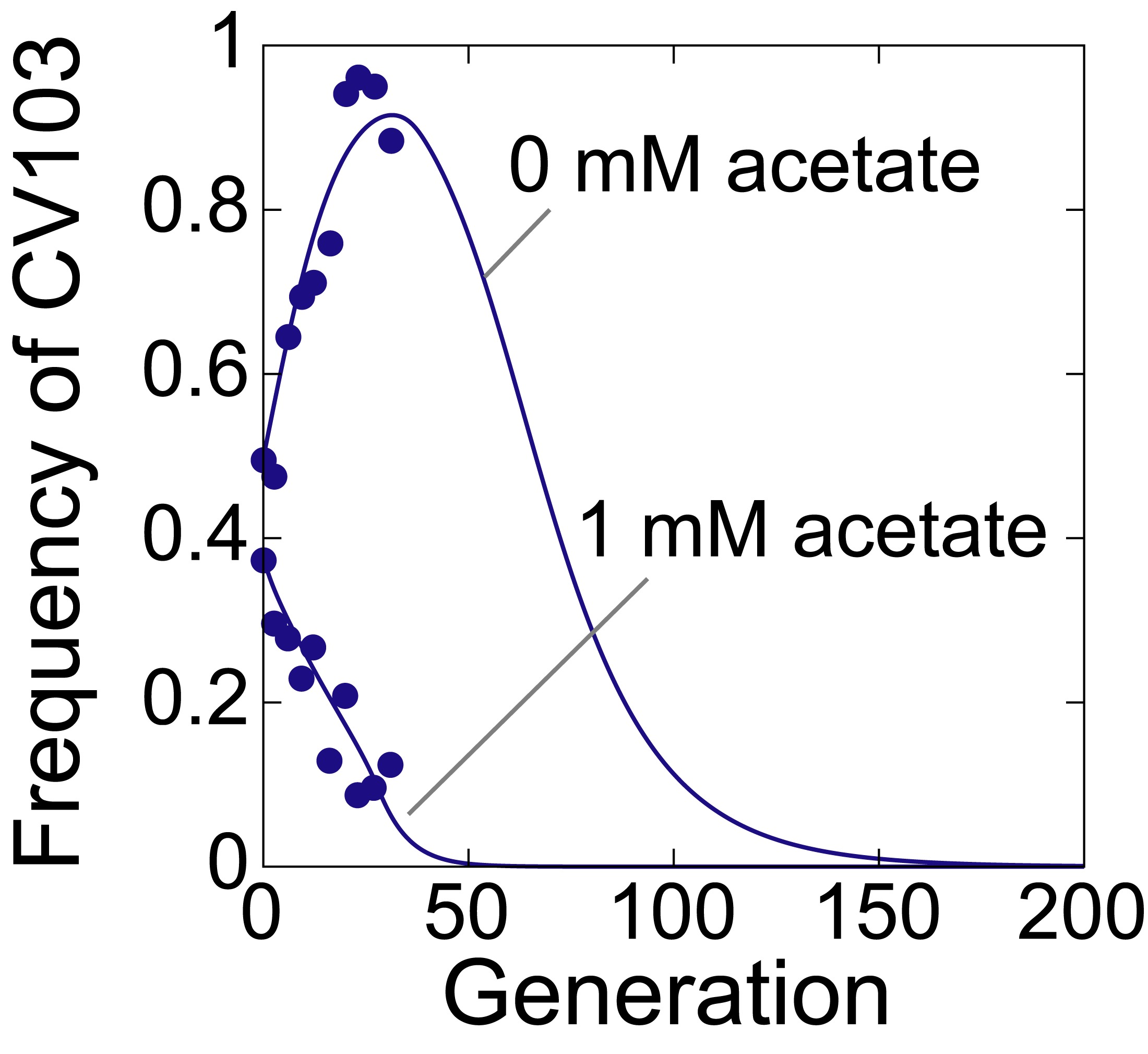

Supplement: S2 Fig — (TIF) [file pcbi.1008135.s002.tif]

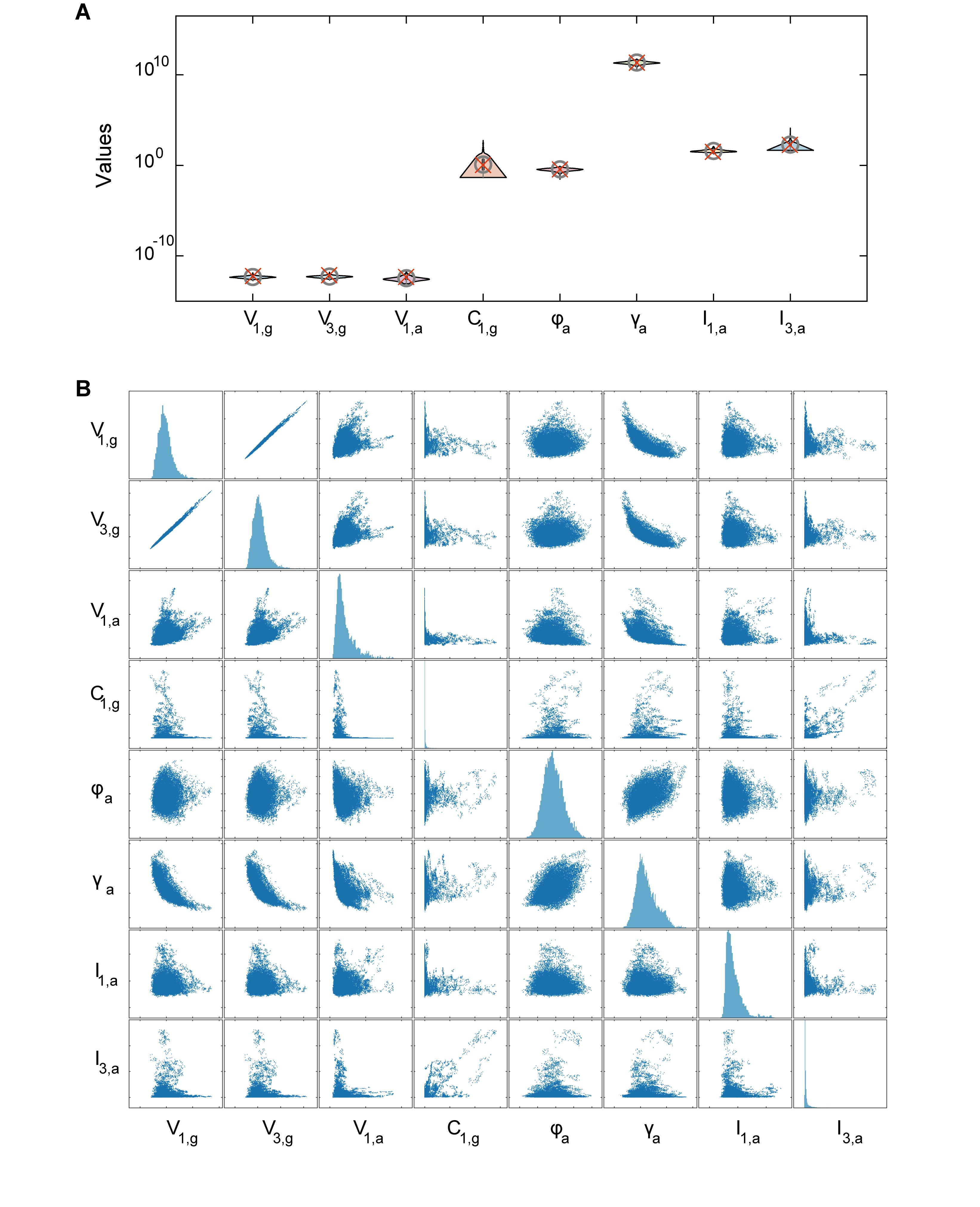

Supplement: S3 Fig — (A) Violin plot of the parameter distributions. Gray circles indicate the median of these distributions and red crosses indicate the values obtained through manual fitting and used in simulations. (B) Pairwise scatter plot of these distributions except that the plots along the diagonal are replaced with histograms of parameter values. Parameters not listed here are either fixed to experimentally measured values or biological constants (see S1 Table for their values). (TIF) [file pcbi.1008135.s003.tif]

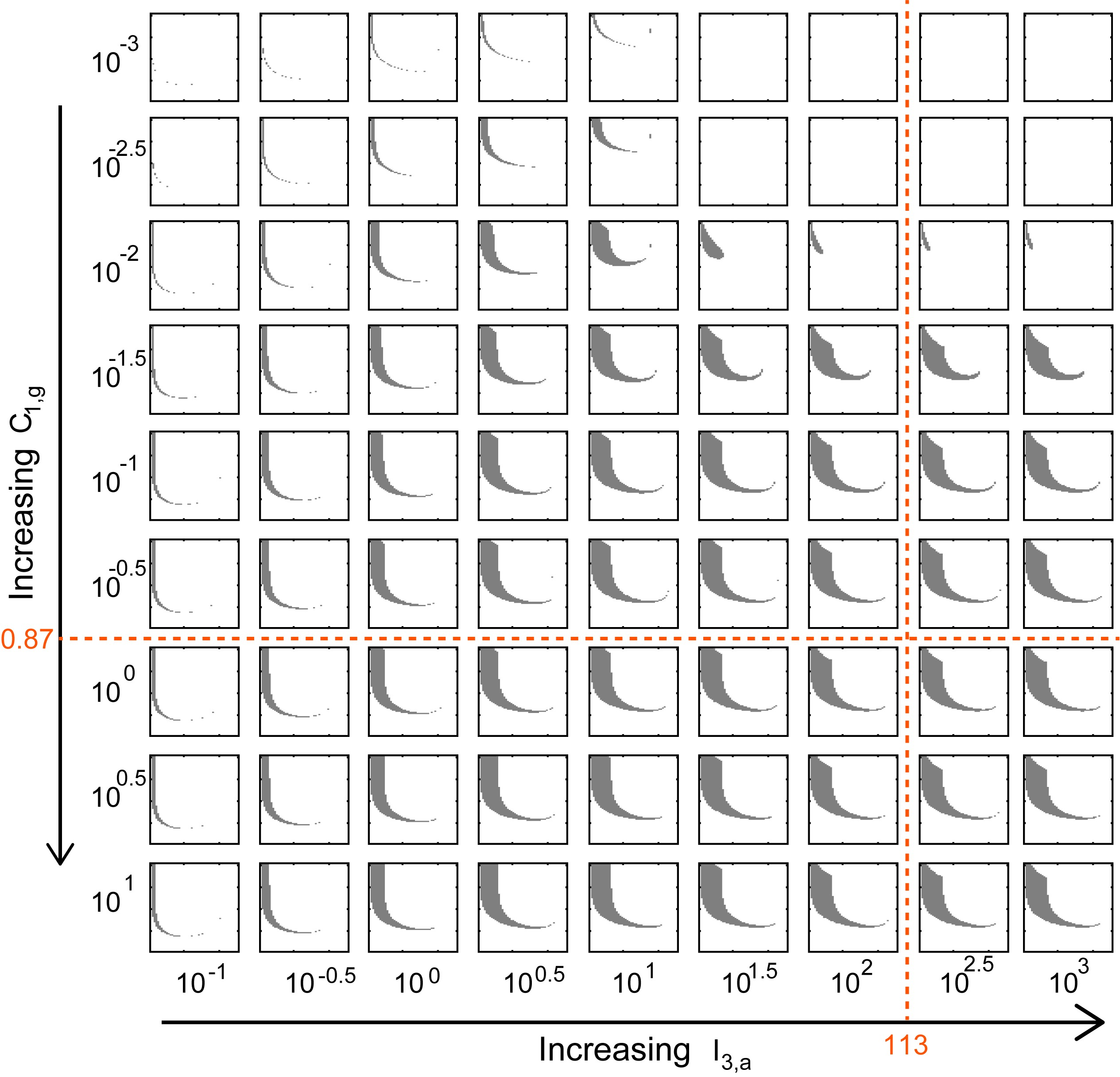

Supplement: S4 Fig — C1,g and I3,a are the parameters that have the largest uncertainty (S3A Fig). Gray shading indicates the region of stable coexistence. The default values of C1,g and I3,a used to generate Fig 2G of the main text are marked by dashed lines. (TIF) [file pcbi.1008135.s004.tif]

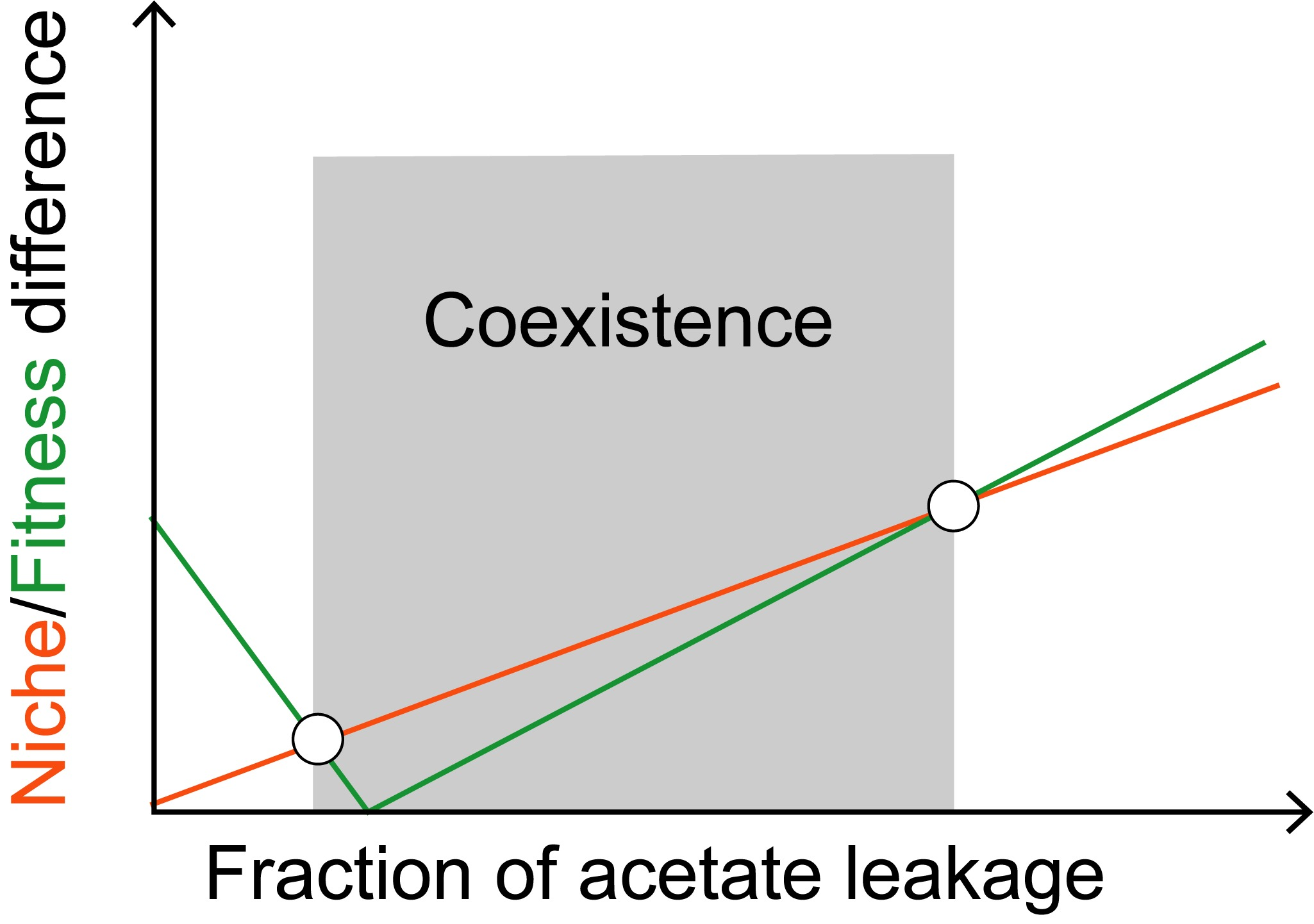

Supplement: S5 Fig — (TIF) [file pcbi.1008135.s005.tif]

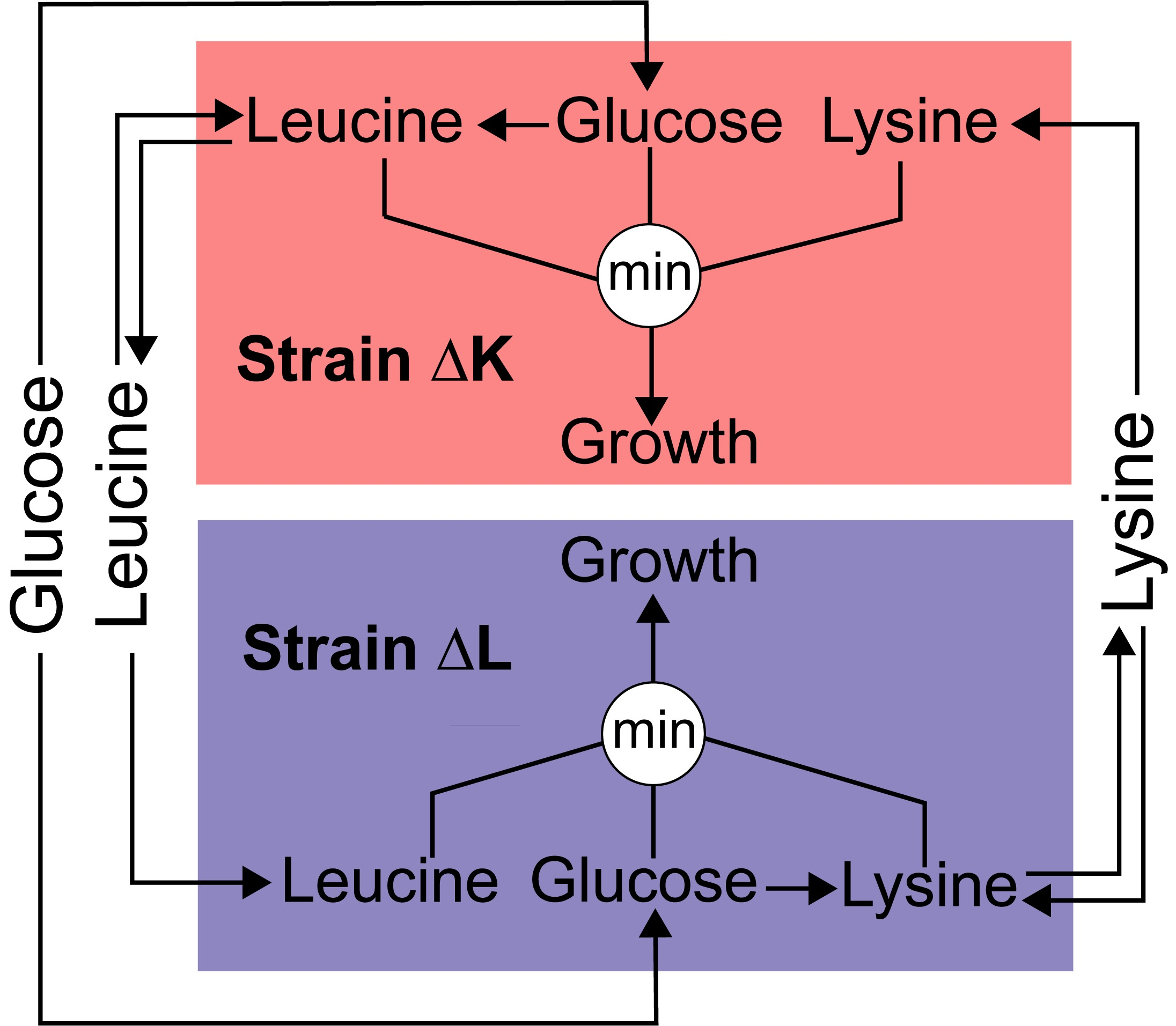

Supplement: S6 Fig — For reference, a simplified version is shown in Fig 3A of the main text. (TIF) [file pcbi.1008135.s006.tif]

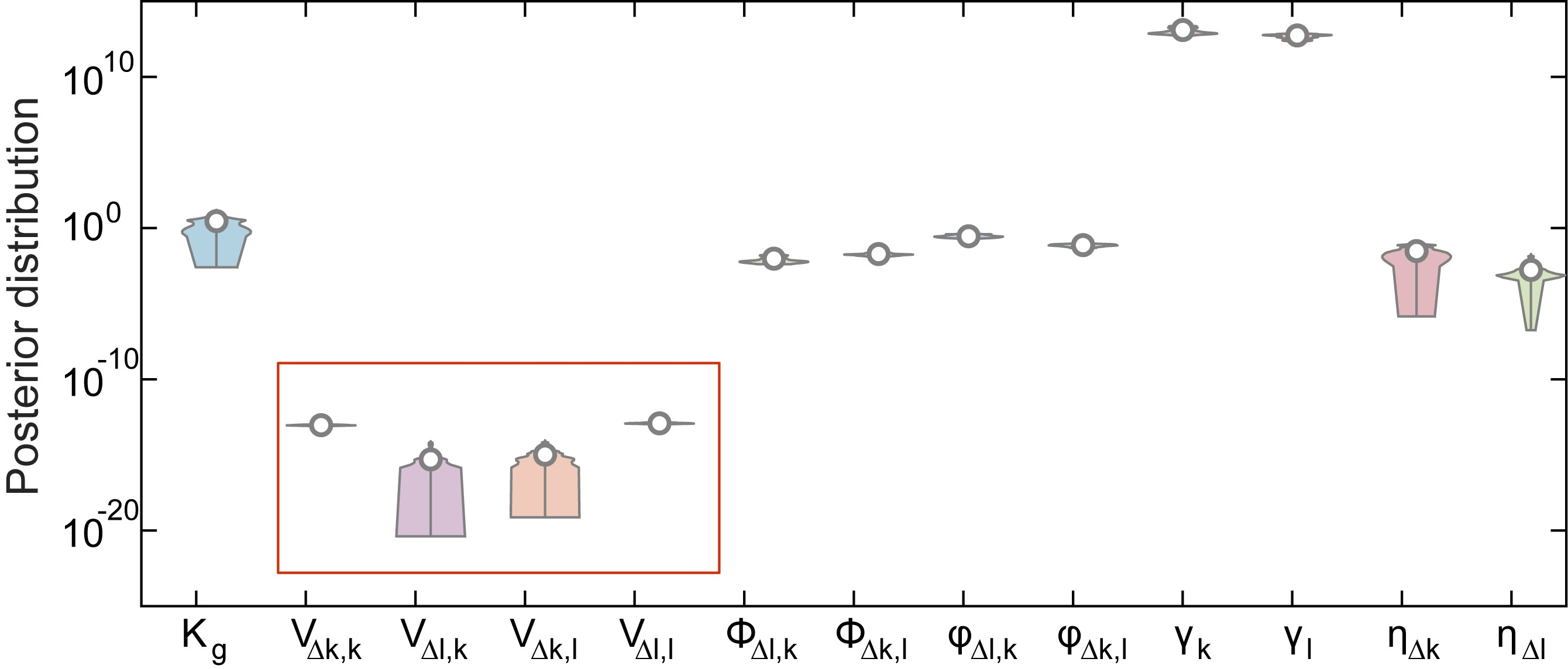

Supplement: S7 Fig — Gray circles indicate the median of these distributions. The red box compares the maximum rates of amino acids uptake by their producing strains (VΔl,k and VΔk,l) and those rates by their non-producing strains (VΔk,k and VΔl,l). Parameters not listed here are either fixed to experimentally measured values or biological constants (see S2 Table for their values). (TIF) [file pcbi.1008135.s007.tif]

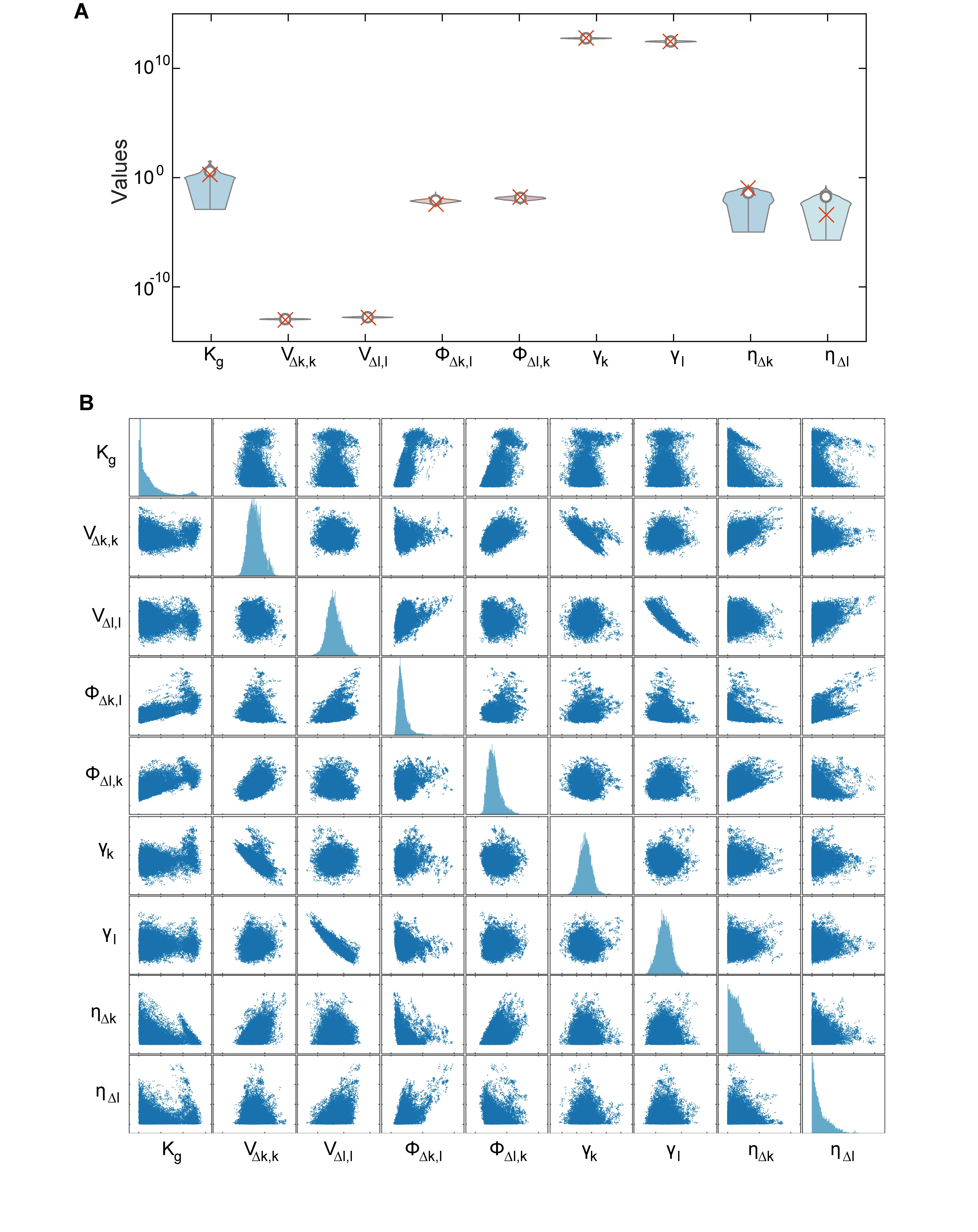

Supplement: S8 Fig — (A) Violin plot of these parameter distributions. Gray circles indicate the median of these distributions and red crosses indicate the values obtained through manual fitting and used in simulations. (B) Pairwise sca_er plot of these distributions except that the plots along the diagonal are replaced with histograms of parameters values. Parameters not listed here are either fixed to experimentally measured values or biological constants (see S2 Table for their values). (TIF) [file pcbi.1008135.s008.tif]

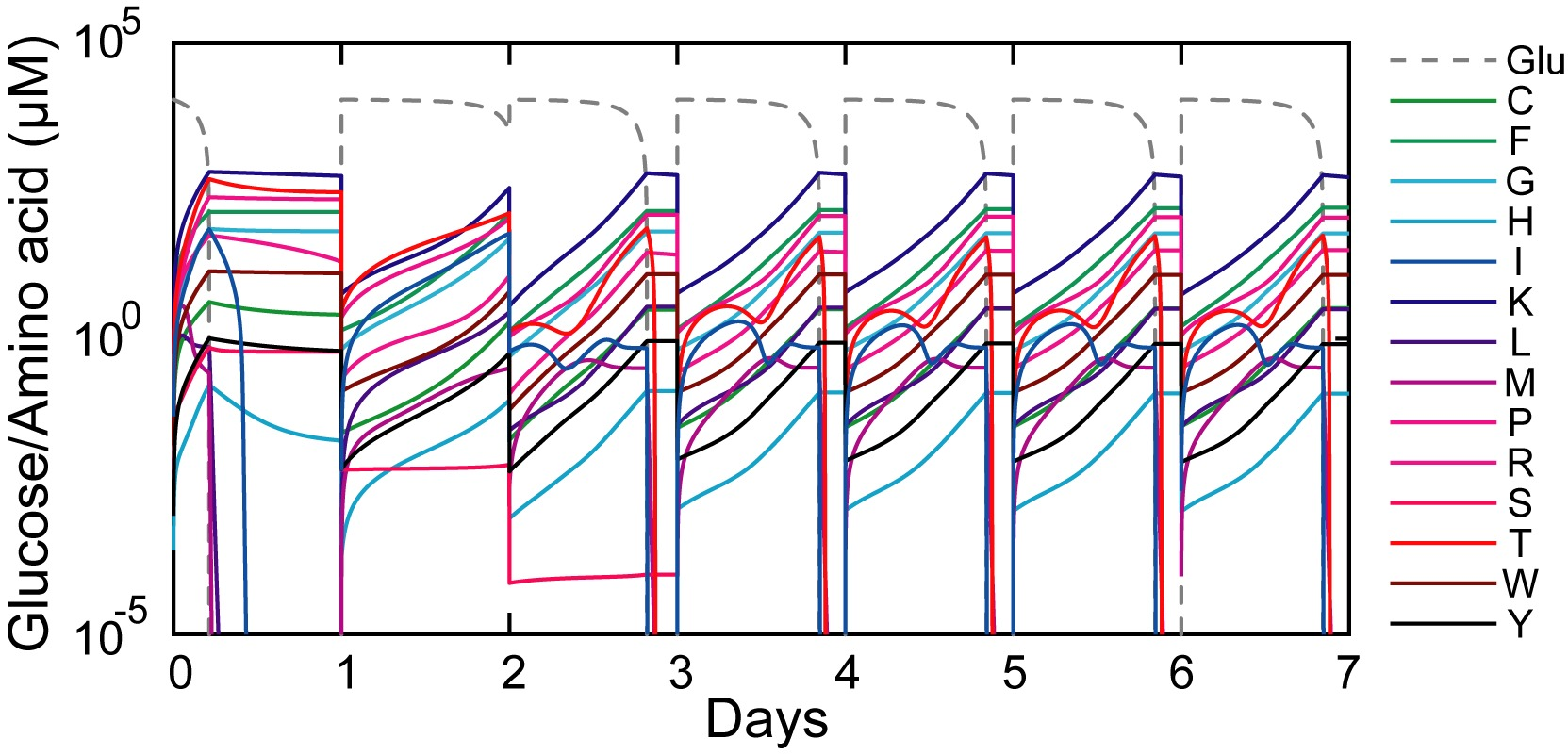

Supplement: S9 Fig — Abbreviations: glucose (Glu), cysteine (C), phenylalanine (F), glycine (G), histidine (H), isoleucine (I), lysine (K), leucine (L), methionine (M), proline (P), arginine (R), serine (S), threonine (T), tryptophan (W), and tyrosine (Y). (TIF) [file pcbi.1008135.s009.tif]

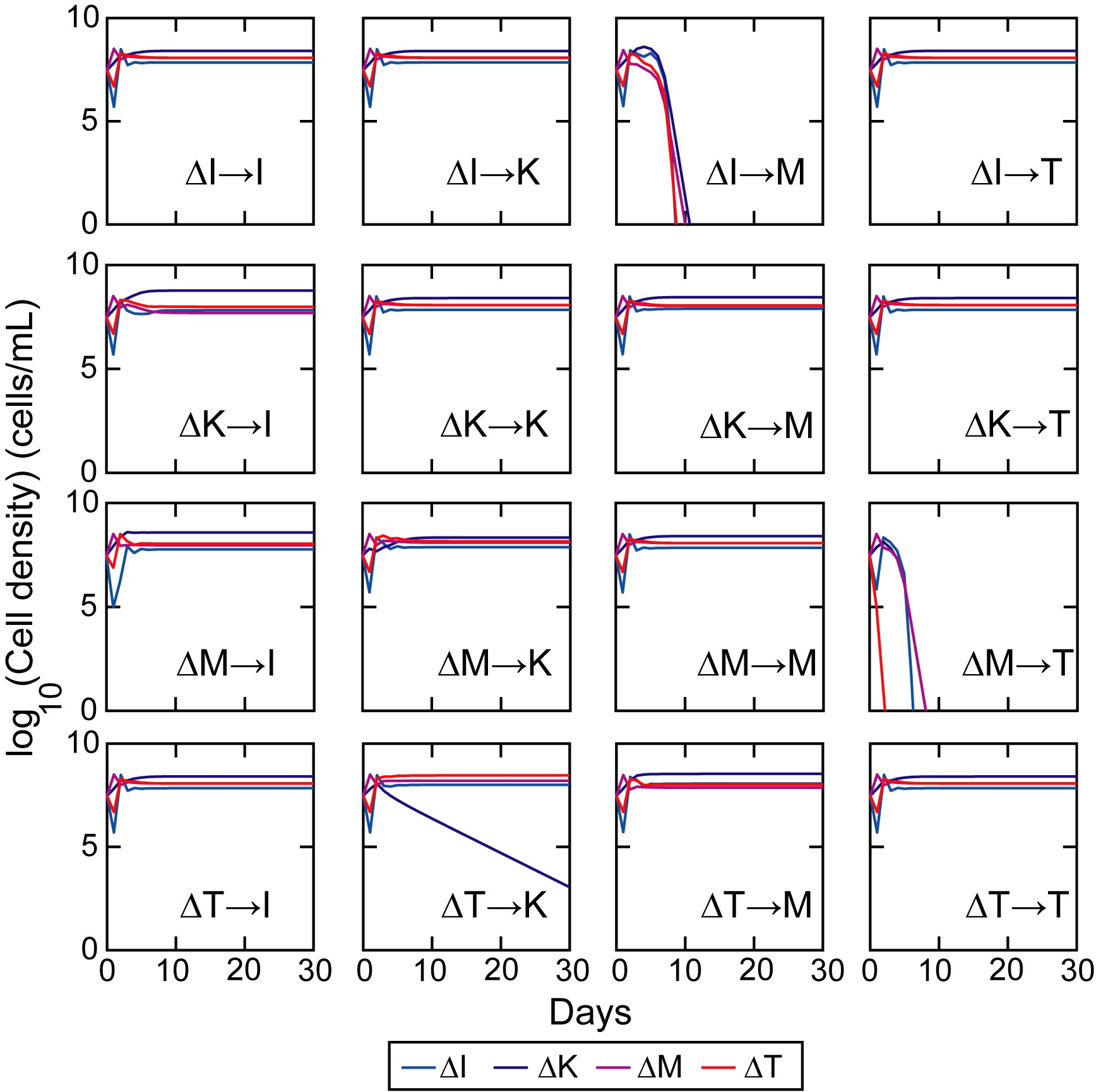

Supplement: S10 Fig — A secretion is deemed as essential if its removal leads to strain loss. Each subplot turns off one secretion reaction: Δx→z indicates the secretion of amino acid z by the amino acid auxotroph Δx. Our simulation results suggest that ΔI→M, ΔM→T, and ΔT→K are essential secretion fluxes. Abbreviations: isoleucine auxotroph (ΔI), lysine auxotroph (ΔK), methionine auxotroph (ΔM), threonine auxotroph (ΔT). (TIF) [file pcbi.1008135.s010.tif]

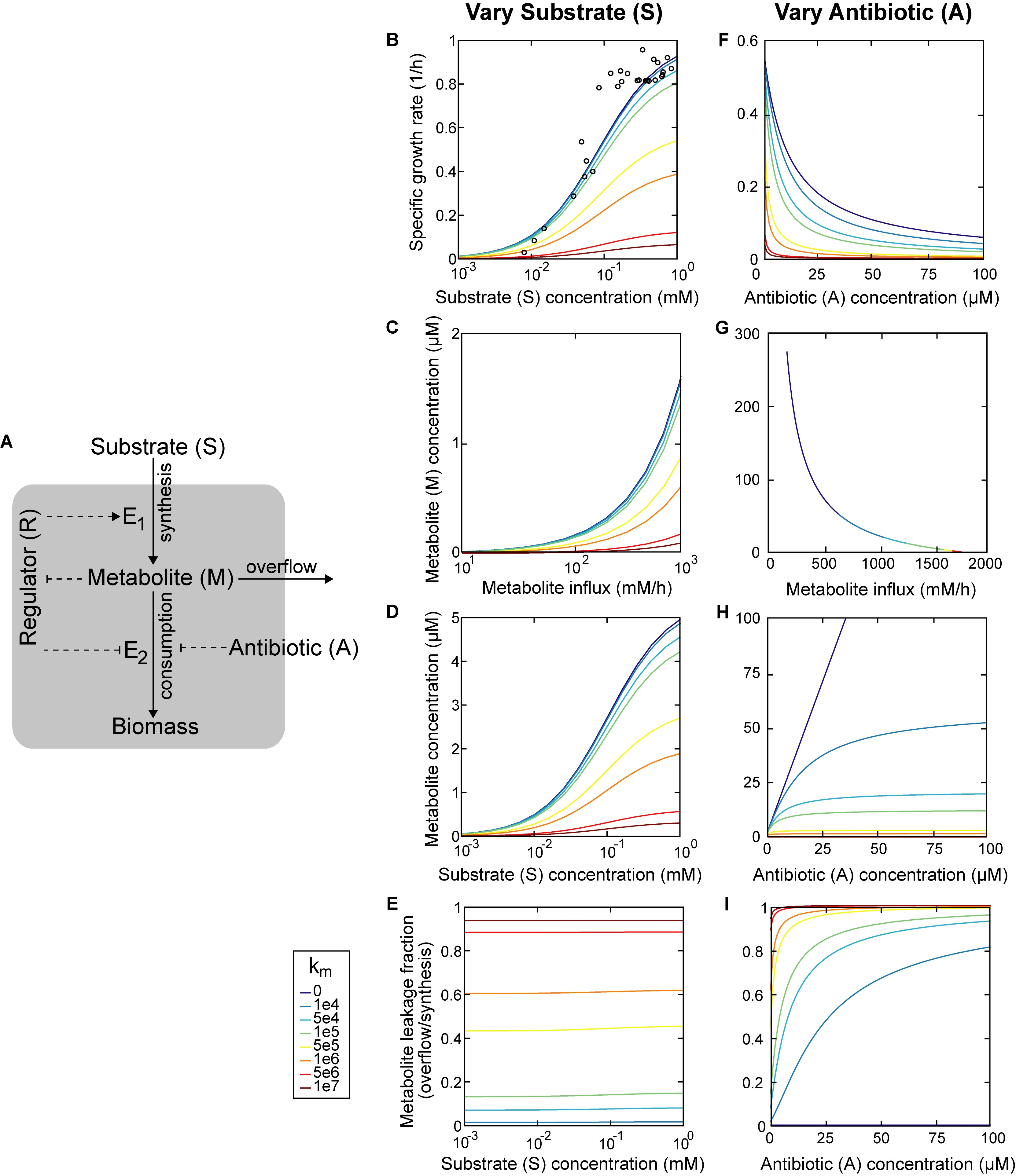

Supplement: S11 Fig — (A) The schematic diagram. E1 and E2 are the enzymes that control biosynthesis and consumption of the metabolite M respectively. Solid point arrows represent material flow. Dashed point arrows represent positive regulations and dashed blunt arrows represent negative regulations. The gray shading represents a cell. (B-E) Steady state values of various quantities by varying external substrate concentration (the antibiotic concentration is 0 μM). In particular, the model reproduces the observed Monod relationship (circles: [57]) in (B) when the diffusion rate constant (km, unit: 1/h) is small. (F-I) Steady state values of the same quantities by varying external antibiotic concentration (the substrate concentration is 100 μM). (TIF) [file pcbi.1008135.s011.tif]
