# Supplementary material for: Modeling microbial cross-feeding at intermediate scale portrays community dynamics and species coexistence
Source: PLoS Comput Biol. 2020 Aug 18;16(8):e1008135. doi: 10.1371/journal.pcbi.1008135 (PMC7480867; doi:10.1371/journal.pcbi.1008135)
Supplement: S1 Table — (PDF) [file pcbi.1008135.s012.pdf]

Parameter	Unit	Default value	Literature	MCMC (Q1)	MCMC (Q2)	MCMC (Q3)	MCMC (95% CI)
$V_{1,g}$	$mmol/h$	5.52×10^{-13}	2.99×10^{-14} (Ref. 1)	3.92×10^{-13}	4.64×10^{-13}	5.48×10^{-13}	$[4.808,\ 4.825]\times10^{-13}$
$V_{3,g}$	$mmol/h$	6.28×10^{-13}	4.43×10^{-14} (Ref. 1)	4.42×10^{-13}	5.24×10^{-13}	6.17×10^{-13}	$[5.427,\ 5.445]\times10^{-13}$
K_g	mM	1.00×10^{-2}	1.00×10^{-2} (Ref. 1, *)				
$I_{1,a}$	mM	2.63×10^{1}	4.00×10^{1} (Ref. 2)	3.31×10^{1}	3.91×10^{1}	4.84×10^{1}	$[4.263,\ 4.281]\times10^{1}$
$I_{3,a}$	mM	1.13×10^{2}	1.00×10^{2} (Ref. 2)	1.39×10^{2}	1.98×10^{2}	3.57×10^{2}	$[4.642,\ 4.772]\times10^{2}$
$C_{1,g}$	mM	8.70×10^{-1}	1.18×10^{1} (Ref. 3)	3.17×10^{-1}	1.23	4.78	$[1.261,\ 1.319]\times10^{1}$
$V_{1,a}$	$mmol/h$	4.26×10^{-13}	1.26×10^{-13} (Ref. 1)	2.51×10^{-13}	3.45×10^{-13}	5.23×10^{-13}	$[4.266.\ 4.299]\times10^{-13}$
$K_{1,a}$	mM	2.00×10^{-1}	2.00×10^{-1} (Ref. 1, *)				
γ_a	$1/mmol$	2.00×10^{11}	$[0.67,\ 1.00]\times10^{11}$ (Ref. 4)	1.90×10^{11}	2.31×10^{11}	2.92×10^{11}	$[2.454,\ 2.463]\times10^{11}$
φ_a		3.30×10^{-1}		2.98×10^{-1}	3.67×10^{-1}	4.46×10^{-1}	$[3.749,\ 3.763]\times10^{-1}$
δ_a		3.00	Constant (*)				

Supplementary Table 1: Estimated parameter values for the simplified unilateral cross-feeding model. The column "Default value" are the parameter values obtained through manual fitting and used in all simulations. The column "Literature" lists values reported in literature. A literature value is marked with “*” if the corresponding parameter is constrained to be equal to this value during parameter fitting processes. The columns "MCMC (Q1)", "MCMC (Q2)", "MCMC (Q3)" and "MCMC (95% CI)" are the 25% percentile, 50% percentile (median), 75% percentile and 95% confidence interval of their posterior distributions sampled by Markov-Chain-Monte-Carlo algorithm. To convert units of $V_{1,g}$, $V_{3,g}$, $V_{1,a}$, and γ_a from their original data, we assume 3×10^{-13} g dry mass per cell. We chose $\delta_a = 3$, which conserves carbon in the production of acetate from glucose. The "carbon conservation" choice may not reflect the actual stoichiometry in cells: one molecule of glucose can also be fermented to produce 2 molecules of acetate and 2 molecules of carbon dioxide (in this case, $\delta_a = 2$). But most importantly, the different choices of δ_a would not alter model behavior because it can be absorbed into $V_{1,g}$ and $V_{3,g}$ as a scaling factor.

References

[1] R Frank Rosenzweig, RR Sharp, David S Treves, and Julian Adams. Microbial evolution in a simple unstructured environment: genetic differentiation in *Escherichia coli*. *Genetics*, 137(4):903–917, 1994.

[2] Ivana Gudelj, Margie Kinnersley, Peter Rashkov, Karen Schmidt, and Frank Rosenzweig. Stability of cross-feeding polymorphisms in microbial communities. *PLoS computational biology*, 12(12):e1005269, 2016.

[3] Emmanuel Anane, Peter Neubauer, M Nicolas Cruz Bournazou, et al. Modelling overflow metabolism in *Escherichia coli* by acetate cycling. *Biochemical Engineering Journal*, 125:23–30, 2017.

[4] Brice Enjalbert, Pierre Millard, Mickael Dinclaux, Jean-Charles Portais, and Fabien Létisse. Acetate fluxes in *Escherichia coli* are determined by the thermodynamic control of the Pta-AckA pathway. *Scientific Reports*, 7:42135, 2017.
