# Supplementary material for: Modeling microbial cross-feeding at intermediate scale portrays community dynamics and species coexistence
Source: PLoS Comput Biol. 2020 Aug 18;16(8):e1008135. doi: 10.1371/journal.pcbi.1008135 (PMC7480867; doi:10.1371/journal.pcbi.1008135)
Supplement: S2 Table — (PDF) [file pcbi.1008135.s013.pdf]

Parameter	Unit	Default value	Literature	MCMC (Q1)	MCMC (Q2)	MCMC (Q3)	MCMC (95% CI)
V_g	$mmol/h$	3.61×10^{-12}	3.61×10^{-12} (Ref. [1], *)				
K_g	mM	1.75	7.00×10^{-2} (Ref. [2])	9.97×10^{-1}	3.00	6.94	[5.388, 5.467]
$V_{\Delta k,k}$	$mmol/h$	8.35×10^{-14}	4.83×10^{-14} (Ref. [3])	8.29×10^{-14}	8.82×10^{-14}	9.43×10^{-14}	$[8.892,\ 8.902]\times10^{-13}$
$K_{\Delta k,k}$	mM	5.00×10^{-3}	5.00×10^{-3} (Ref. [4], *)				
$V_{\Delta l,l}$	$mmol/h$	1.22×10^{-13}	6.60×10^{-14} (Ref. [3])	1.23×10^{-13}	1.31×10^{-13}	1.41×10^{-13}	$[1.322,\ 1.323]\times10^{-13}$
$K_{\Delta l,l}$	mM	1.07×10^{-3}	1.07×10^{-3} (Ref. [5], *)				
γ_g	$1/mmol$	3.00×10^{11}	3.00×10^{11} (Ref. [6], *)				
γ_k	$1/mmol$	5.72×10^{12}	9.52×10^{12} (Ref. [3])	4.31×10^{12}	4.58×10^{12}	4.86×10^{12}	$[4.594,\ 4.599]\times10^{12}$
γ_l	$1/mmol$	2.53×10^{12}	7.05×10^{12} (Ref. [3])	2.16×10^{12}	2.34×10^{12}	$2.53\times10^{!2}$	$[2.351,\ 2.354]\times10^{12}$
$\varphi_{\Delta k,l}$		3.20×10^{-3}		5.20×10^{-3}	6.60×10^{-3}	8.50×10^{-3}	$[7.400,\ 7.500]\times10^{-3}$
$\varphi_{\Delta l,k}$		1.39×10^{-2}		9.10×10^{-3}	1.13×10^{-2}	1.41×10^{-2}	$[1.199,\ 1.204]\times10^{-2}$
δ_k		1.00	Constant (*)				
δ_l		1.00	Constant (*)				
$\eta_{\Delta k}$	$1/h$	1.00×10^{-1}	7.32×10^{-2} (Ref. [3])	1.56×10^{-2}	3.29×10^{-2}	5.61×10^{-2}	$[3.850,\ 3.880]\times10^{-2}$
$\eta_{\Delta l}$	$1/h$	4.00×10^{-4}	1.00×10^{-4} (Ref. [3])	5.40×10^{-3}	1.41×10^{-2}	3.08×10^{-2}	$[2.080,\ 2.110]\times10^{-2}$

Supplementary Table 2: Estimated parameter values for the simplified bilateral cross-feeding model. The column "Default value" are the parameter values obtained through manual fitting and used in all simulations. The column "Literature" lists values reported in literature. A literature value is marked with "*" if the corresponding parameter is constrained to be equal to this value during parameter fitting processes. The columns "MCMC (Q1)", "MCMC (Q2)", "MCMC (Q3)" and "MCMC (95% CI)" are the 25% percentile, 50% percentile (median), 75% percentile and 95% confidence interval of their posterior distributions sampled by Markov-Chain-Monte-Carlo algorithm. To convert unit of V_g, $V_{\Delta k,k}$, $V_{\Delta l,l}$, γ_g, γ_k and γ_l from original data, we assume 3×10^{-13} g dry mass per cell. $K_{\Delta k,k}$ was calculated as the geometric mean of Km values of two active lysine transport systems. We chose $\delta_k = \delta_l = 1$ to conserve carbon in the production of lysine and leucine from glucose.

References

[1] Rishi Jain and Ranjan Srivastava. Metabolic investigation of host/pathogen interaction using MS2-infected *Escherichia coli*. *BMC Systems Biology*, 3(1):121, 2009.

[2] Arijit Maitra and Ken A Dill. Bacterial growth laws reflect the evolutionary importance of energy efficiency. *Proc. Natl. Acad. Sci. USA*, 112(2):406–411, 2015.

[3] Xiaolin Zhang and Jennifer L Reed. Adaptive evolution of synthetic cooperating communities improves growth performance. *PLoS ONE*, 9(10):e108297, 2014.

[4] Yeheskel S Halpern. Genetics of amino acid transport in bacteria. *Annual Review of Genetics*, 8(1):103–133, 1974.

[5] Jeanette R Piperno and Dale L Oxender. Amino acid transport systems in *Escherichia coli* K12. *Journal of Biological Chemistry*, 243(22):5914–5920, 1968.

[6] Joseph Shiloach and Rephael Fass. Growing *E. coli* to high cell density–a historical perspective on method development. *Biotechnology Advances*, 23(5):345–357, 2005.
