# Supplementary material for: Modeling microbial cross-feeding at intermediate scale portrays community dynamics and species coexistence
Source: PLoS Comput Biol. 2020 Aug 18;16(8):e1008135. doi: 10.1371/journal.pcbi.1008135 (PMC7480867; doi:10.1371/journal.pcbi.1008135)
Supplement: S3 Table — (PDF) [file pcbi.1008135.s014.pdf]

Parameter	Unit	Default value	Literature
γ_c	$1/\mu mol$	2.74×10^{9}	2.74×10^{9} (Ref. 1, *)
γ_f	$1/\mu mol$	1.63×10^{10}	1.63×10^{10} (Ref. 1, *)
γ_g	$1/\mu mol$	1.04×10^{9}	1.04×10^{9} (Ref. 1, *)
γ_h	$1/\mu mol$	1.94×10^{10}	1.94×10^{10} (Ref. 1, *)
γ_i	$1/\mu mol$	8.03×10^{9}	8.03×10^{9} (Ref. 1, *)
γ_k	$1/\mu mol$	5.47×10^{9}	5.47×10^{9} (Ref. 1, *)
γ_l	$1/\mu mol$	4.63×10^{9}	4.63×10^{9} (Ref. 1, *)
γ_m	$1/\mu mol$	1.47×10^{10}	1.47×10^{10} (Ref. 1, *)
γ_p	$1/\mu mol$	1.37×10^{9}	1.37×10^{9} (Ref. 1, *)
γ_r	$1/\mu mol$	6.02×10^{9}	6.02×10^{9} (Ref. 1, *)
γ_s	$1/\mu mol$	3.76×10^{8}	3.76×10^{8} (Ref. 1, *)
γ_t	$1/\mu mol$	2.01×10^{9}	2.01×10^{9} (Ref. 1, *)
γ_w	$1/\mu mol$	4.01×10^{10}	4.01×10^{10} (Ref. 1, *)
γ_y	$1/\mu mol$	1.63×10^{10}	1.63×10^{10} (Ref. 1, *)
$V_{\Delta c,c}$	$\mu mol/h$	2.10×10^{-11}	2.10×10^{-11} (Ref. 1, *)
$V_{\Delta f,f}$	$\mu mol/h$	7.00×10^{-12}	7.00×10^{-12} (Ref. 1, *)
$V_{\Delta g,g}$	$\mu mol/h$	1.02×10^{-10}	1.02×10^{-10} (Ref. 1, *)
$V_{\Delta h,h}$	$\mu mol/h$	4.00×10^{-12}	4.00×10^{-12} (Ref. 1, *)
$V_{\Delta i,i}$	$\mu mol/h$	1.15×10^{-10}	1.15×10^{-10} (Ref. 2, *)
$V_{\Delta k,k}$	$\mu mol/h$	5.00×10^{-11}	4.83×10^{-14} (Ref. 3)
$V_{\Delta l,l}$	$\mu mol/h$	1.90×10^{-10}	1.90×10^{-10} (Ref. 2, *)
$V_{\Delta m,m}$	$\mu mol/h$	1.76×10^{-10}	4.68×10^{-11} (Ref. 2)
$V_{\Delta p,p}$	$\mu mol/h$	9.60×10^{-11}	9.60×10^{-11} (Ref. 1, *)
$V_{\Delta r,r}$	$\mu mol/h$	3.20×10^{-11}	$3,69\times10^{-11}$ (Ref. 4)
$V_{\Delta s,s}$	$\mu mol/h$	2.80×10^{-10}	2.80×10^{-10} (Ref. 1, *)
$V_{\Delta t,t}$	$\mu mol/h$	2.79×10^{-10}	1.41×10^{-10} (Ref. 5)
$V_{\Delta w,w}$	$\mu mol/h$	3.00×10^{-12}	3.00×10^{-12} (Ref. 1, *)
$V_{\Delta y,y}$	$\mu mol/h$	4.00×10^{-12}	4.00×10^{-12} (Ref. 1, *)
$K_{\Delta c,c}$	μM	4.96×10^{-1}	4.96×10^{-1} (Ref. 6, *)
$K_{\Delta f,f}$	μM	7.20×10^{-1}	7.20×10^{-1} (Ref. 2, *)
$K_{\Delta g,g}$	μM	3.80	3.80 (Ref. 2, *)
$K_{\Delta h,h}$	μM	2.60×10^{-2}	1.00 (Ref. 4)
$K_{\Delta i,i}$	μM	2.20×10^{-1}	1.22 (Ref. 2)
$K_{\Delta k,k}$	μM	5.00	5.00 (Ref. 4, *)
$K_{\Delta l,l}$	μM	1.07	1.07 (Ref. 2, *)
$K_{\Delta m,m}$	μM	2.27	2.27 (Ref. 2, *)
$K_{\Delta p,p}$	μM	2.00	2.00 (Ref. 7, *)
$K_{\Delta r,r}$	μM	5.00×10^{-2}	2.60×10^{-2} (Ref. 4)
$K_{\Delta s,s}$	μM	7.50×10^{-1}	8.95 (Ref. 8)
$K_{\Delta t,t}$	μM	5.40×10^{-1}	3.90×10^{-1} (Ref. 5)
$K_{\Delta w,w}$	μM	9.00×10^{-1}	9.00×10^{-1} (Ref. 2, *)
$K_{\Delta y,y}$	μM	3.40×10^{-1}	3.40×10^{-1} (Ref. 9, *)
$\eta_{\Delta c}$	$1/h$	1.50×10^{-1}	
$\eta_{\Delta f}$	$1/h$	3.00×10^{-1}	

$\eta_{\Delta g}$	$1/h$	2.00×10^{-1}	
$\eta_{\Delta h}$	$1/h$	2.00×10^{-1}	
$\eta_{\Delta i}$	$1/h$	4.00×10^{-1}	
$\eta_{\Delta k}$	$1/h$	0.00	7.32×10^{-2} (Ref. 3)
$\eta_{\Delta l}$	$1/h$	2.00×10^{-1}	1.00×10^{-4} (Ref. 3)
$\eta_{\Delta m}$	$1/h$	0.00	
$\eta_{\Delta p}$	$1/h$	1.00×10^{-1}	
$\eta_{\Delta r}$	$1/h$	0.00	
$\eta_{\Delta s}$	$1/h$	7.50×10^{-1}	
$\eta_{\Delta t}$	$1/h$	2.00×10^{-1}	
$\eta_{\Delta w}$	$1/h$	5.00×10^{-2}	
$\eta_{\Delta y}$	$1/h$	1.00×10^{-1}	
δ_c		2.00	Constant (*)
δ_f		0.67	Constant (*)
δ_g		3.00	Constant (*)
δ_h		1.00	Constant (*)
δ_i		1.00	Constant (*)
δ_k		1.00	Constant (*)
δ_l		1.00	Constant (*)
δ_m		1.20	Constant (*)
δ_p		1.20	Constant (*)
δ_r		1.00	Constant (*)
δ_s		2.00	Constant (*)
δ_t		1.50	Constant (*)
δ_w		0.55	Constant (*)
δ_y		0.67	Constant (*)
V_g	$\mu mol/h$	3.61×10^{-9}	3.61×10^{-9}(Ref. 10, *)
K_g	μM	1.75	1.75 (Ref. 11, *)
γ_g	$1/\mu mol$	3.00×10^{8}	3.00×10^{8} (Ref. 12, *)

Supplementary Table 3: Estimated parameter values for the multilateral cross-feeding model. The default values of the amino acid leakage fractions ($\varphi_{\Delta x,z}$, x, $z \in \{c, f, g, h, i, k, l, m, p, r, s, t, w, y\}$) are displayed in the main text Fig. 5C. To convert unit of V_g and γ_g from original data, we assume 3×10^{-13} g dry mass per cell. To convert unit of $V_{\Delta x,x}$ ($x \in \{i, k, l, m, r, t\}$) from original data, we assume 1×10^{-12} g wet mass per cell and 0.2 pg protein per cell. The other $V_{\Delta x,x}$ ($x \in \{c, f, g, h, p, s, w, y\}$) values were directly estimated from the measured growth rates [1] of corresponding auxotrophies by multiplying the biomass yields of *E. coli* on these amino acids. $K_{\Delta k,k}$ and $K_{\Delta h,h}$ were calculated as the geometric mean of Km values of two active lysine and histidine transport systems respectively. The values of δ_x ($x \in \{c, f, g, h, i, k, l, m, p, r, s, t, w, y\}$) were chosen to conserve carbon in the production of amino acids from glucose.

References

[1] Michael T Mee, James J Collins, George M Church, and Harris H Wang. Syntrophic exchange in synthetic microbial communities. *Proceedings of the National Academy of Sciences*, 111(20):E2149–E2156, 2014.

[2] Jeanette R Piperno and Dale L Oxender. Amino acid transport systems in *Escherichia coli* K12. *Journal of Biological Chemistry*, 243(22):5914–5920, 1968.

[3] Xiaolin Zhang and Jennifer L Reed. Adaptive evolution of synthetic cooperating communities improves growth performance. *PLoS ONE*, 9(10):e108297, 2014.

[4] Yeheskel S Halpern. Genetics of amino acid transport in bacteria. *Annual Review of Genetics*, 8(1):103–133, 1974.

[5] Bonnie A Templeton and Michael A Savageau. Transport of biosynthetic intermediates: homoserine and threonine uptake in *Escherichia coli. Journal of bacteriology*, 117(3):1002–1009, 1974.

[6] Iwao Ohtsu, Yusuke Kawano, Marina Suzuki, Susumu Morigasaki, Kyohei Saiki, Shunsuke Yamazaki, Gen Nonaka, and Hiroshi Takagi. Uptake of L-cystine via an ABC transporter contributes defense of oxidative stress in the L-cystine export-dependent manner in *Escherichia coli. PLoS ONE*, 10(4):e0120619, 2015.

[7] Robert Landick, Dale L Oxender, and Giovanna Ferro-Luzzi Ames. Bacterial amino acid transport systems. In *The Enzymes of Biological Membranes*, pages 577–615. Springer, 1985.

[8] Sharon D Cosloy. D-serine transport system in *Escherichia coli* k-12. *Journal of bacteriology*, 114(2):679–684, 1973.

[9] Matthias Quick and Jonathan A Javitch. Monitoring the function of membrane transport proteins in detergent-solubilized form. *Proceedings of the National Academy of Sciences*, 104(9):3603–3608, 2007.

[10] Rishi Jain and Ranjan Srivastava. Metabolic investigation of host/pathogen interaction using MS2-infected *Escherichia coli. BMC Systems Biology*, 3(1):121, 2009.

[11] Arvind Natarajan and Friedrich Srienc. Dynamics of glucose uptake by single *Escherichia coli* cells. *Metabolic Engineering*, 1(4):320–333, 1999.

[12] Joseph Shiloach and Rephael Fass. Growing *E. coli* to high cell density–a historical perspective on method development. *Biotechnology Advances*, 23(5):345–357, 2005.
