# Supplementary material for: Modeling microbial cross-feeding at intermediate scale portrays community dynamics and species coexistence
Source: PLoS Comput Biol. 2020 Aug 18;16(8):e1008135. doi: 10.1371/journal.pcbi.1008135 (PMC7480867; doi:10.1371/journal.pcbi.1008135)
Supplement: S4 Table — (PDF) [file pcbi.1008135.s015.pdf]

Parameter	Unit	Value	Source
α	μM	3.00×10^6	Ref. [1]
$k_{e,1}$	$1/h$	1.20×10^3	
$k_{e,2}$	$1/h$	7.56×10^4	Ref. [1]
$m_{e,1}$		325	Ref. [2]
$m_{e,2}$		11738	Ref. [3]
$K_{m,s}$	μM	1.00×10^2	
$K_{m,m}$	μM	2.00×10^1	Ref. [1]
$K_{i,r}$	μM	6.00×10^1	
k_r	$1/h$	3.60×10^3	Ref. [1]
d_r	$1/h$	1.26×10^2	Ref. [1]
Y_m	$1/\mu M$	3.33×10^{-7}	
M_e	μM	0.00	
$K_{i,a}$	μM	1.00	

Supplementary Table 4: Parameter values used in the simulation of the single-strain model. Note that these values are specific to *E. coli*. The value of $m_{e,2}$ was calculated by multiplying the literature value (7336 amino acids in ribosomal proteins) by a factor of 1.6 to account for tRNA-affiliated proteins. The yield coefficient of amino acid is the inverse of the total amino acid concentration of an *Escherichia coli* cell, i.e., $Y_m = 1/\alpha$. By choosing $M_e = 0$, we assume that the metabolite released to the environment does not accumulate and can be quickly metabolized by other cell types.

References

[1] Allen G Marr. Growth rate of *Escherichia coli*. *Microbiol. Rev.*, 55(2):316–333, 1991.

[2] Arijit Maitra and Ken A Dill. Bacterial growth laws reflect the evolutionary importance of energy efficiency. *Proc. Natl. Acad. Sci. USA*, 112(2):406–411, 2015.

[3] Stefan Klumpp, Matthew Scott, Steen Pedersen, and Terence Hwa. Molecular crowding limits translation and cell growth. *Proc. Natl. Acad. Sci. USA*, 110(42):16754–16759, 2013.
