# Supplementary material for: Modeling microbial cross-feeding at intermediate scale portrays community dynamics and species coexistence
Source: PLoS Comput Biol. 2020 Aug 18;16(8):e1008135. doi: 10.1371/journal.pcbi.1008135 (PMC7480867; doi:10.1371/journal.pcbi.1008135)
Supplement: S1 Text — (PDF) [file pcbi.1008135.s016.pdf]

Supporting Information

Chen Liao, Tong Wang, Sergei Maslov, Joao B. Xavier

July 31, 2020

Contents

1 A Biophysical Modeling Framework for Microbial Community **2**

2 Unilateral Cross-Feeding between Glucose and Acetate Specialists **6**
2.1 Model Specification from the General Framework 6
2.2 Simplifying Assumptions and Justifications 7

3 Bilateral Cross-Feeding between Lysine and Leucine auxotrophies **9**
3.1 Model Specification from the General Framework 9
3.2 Simplifying Assumptions and Justifications 10

4 Multilateral Cross-Feeding between 14 Amino Acid auxotrophies **12**
4.1 Model Specification from the General Framework 12
4.2 Simplified Pairwise Batch Co-culture Model 13

5 Discussions on the Proportionality Assumption for Leakage Rate **14**

6 References **17**

1 A Biophysical Modeling Framework for Microbial Community

In this study, we present a biophysical model that combines population dynamics of different cell types that complete for external nutrients and their intracellular metabolism. The population dynamics part of our model (i.e., cell population expansion and contraction) is similar to previous models [1, 2, 3] but the part with respect to the intracellular metabolism is very different. We considered the internal metabolism of nutrient uptake and conversion via a simplified 3-level metabolic network that captures resource transformation from growth substrates to metabolic building blocks and then to biomass. Growth substrates, either substitutable or non-substitutable, are molecules that are able to support cell growth as the sole sources that supply nutrients they contain. Metabolic building blocks are incorporated into different functional units of biomass and thus assume to be non-substitutable. Note that in our coarse-grained picture, building blocks can be any metabolite intermediates that serve as precursors of molecules that are actually integrated into biomass. In some cases, a molecule can be a growth substrate for one cell type and a metabolite precursor for another.

The variables we considered in our model include: (1) Density of active cells of n_c microbial populations with distinct cell types (N_l, $l = 1, 2, \cdots, n_c$); (2) Concentration of n_s growth substrates in the culture medium (S_i, $i = 1, 2, \cdots, n_s$) and in cell type l ($\hat{S}_{l,i}$, $i = 1, 2, \cdots, n_s$); (3) Concentration of n_m metabolic building blocks in the culture medium (M_j, $j = 1, 2, \cdots, n_m$) and in cell type l ($\hat{M}_{l,j}$, $j = 1, 2, \cdots, n_m$). The biochemical reactions that define the relationships among these variables are given below

$$S_i \xrightarrow{J_{l,i}^{upt,S}} \hat{S}_{l,i} \quad l = 1, 2, \cdots, n_c, \;\; i = 1, 2, \cdots, n_s \tag{1}$$

$$M_j \xrightarrow{J_{l,j}^{upt,M}} \hat{M}_{l,j} \quad l = 1, 2, \cdots, n_c, \;\; j = 1, 2, \cdots, n_m \tag{2}$$

$$\sum_{i=1}^{n_s} (1/\delta_{l,i,j}) \hat{S}_{l,i} \xrightarrow{J_{l,j}^{syn}} \hat{M}_{l,j} \quad l = 1, 2, \cdots, n_c, \;\; j = 1, 2, \cdots, n_m \tag{3}$$

$$\hat{M}_{l,j} \xrightarrow{J_{l,j}^{leak}} M_j \quad l = 1, 2, \cdots, n_c, \;\; j = 1, 2, \cdots, n_m \tag{4}$$

$$\sum_{i=1}^{n_s} (1/\gamma_{l,i}) \hat{S}_{l,i} + \sum_{j=1}^{n_m} (1/\gamma_{l,j}) \hat{M}_{l,j} \xrightarrow{J_{l}^{grow}} \text{An active cell of population } l \quad l = 1, 2, \cdots, n_c \tag{5}$$

$$\text{An active cell of population } l \xrightarrow{J_{l}^{death}} \text{An inactive cell of population } l \quad l = 1, 2, \cdots, n_c \tag{6}$$

$$\hat{S}_{l,i} \xrightarrow{J_{l,i}^{sink,S}} \emptyset \quad l = 1, 2, \cdots, n_c, \;\; i = 1, 2, \cdots, n_s \tag{7}$$

$$\hat{M}_{l,j} \xrightarrow{J_{l,j}^{sink,M}} \emptyset \quad l = 1, 2, \cdots, n_c, \;\; j = 1, 2, \cdots, n_m \tag{8}$$

where J's represents reaction rates (fluxes). Equation (1) and (2) describe resource uptake into intracellular space. Equation (3) describes biosynthesis of intermediate metabolites $\hat{M}_{l,j}$ from substrates $\hat{S}_{l,i}$, where $1/\delta_{l,i,j}$ is the number of molecules of substrate $\hat{S}_i$ consumed for every one molecule of metabolite $\hat{M}_j$ produced by cell type l. Note that Equation (3) is a lumped and elementary form of realistic metabolic reactions which allow multiple products on the right-hand side and also multiple cooccuring reactions with the same reactants/products but different stoichiometry. Equation (4) describes the leakage of intracellular metabolites $\hat{M}_{l,j}$ into the environment. Equation (5) describes biomass synthesis from both substrate $\hat{S}_{l,i}$ and metabolic building blocks $\hat{M}_{l,j}$, where $\gamma_{l,i}$ and $\gamma_{l,j}$ are biomass yield coefficients (alternatively, $1/\gamma_{l,i}$ and $1/\gamma_{l,j}$ represent the numbers of molecules of $\hat{S}_{l,i}$ and $\hat{M}_{l,j}$ needed to construct one active cell of cell type l respectively). Importantly, the first sum over substrates in Equation (5) approximates biomass composition from other building blocks that are not explicitly modeled. Equation (6) describes a decrease

in viable biomass, i.e., cell death. Equation (7) and (8) represent sink reactions that consume substrates $\hat{S}_{l,i}$ and metabolites $\hat{M}_{l,j}$ through non-growth metabolic processes and degradation pathways.

We consider constant supply rate of growth substrates in a chemostat culture with dilution rate D. The eight reactions shown above can be translated to the following differential equations

$$\frac{d[S_i]}{dt} = D(S_{0,i} - [S_i]) - \sum_{l=1}^{n_c} J_{l,i}^{upt,S} N_l \qquad i = 1, 2, \cdots, n_s \tag{9}$$

$$\frac{dN_l}{dt} = N_l \left(J_l^{grow} - J_l^{death} - D \right) \qquad l = 1, 2, \cdots, n_c \tag{10}$$

$$\frac{d[M_j]}{dt} = D(M_{0,j} - [M_j]) + \sum_{l=1}^{n_c} \left(J_{l,j}^{leak} - J_{l,j}^{upt,M} \right) N_l \qquad j = 1, 2, \cdots, n_m \tag{11}$$

$$\frac{d[\hat{S}_{l,i}]}{dt} = J_{l,i}^{upt,S} - \sum_{j=1}^{n_m} (1/\delta_{l,i,j}) J_{l,j}^{syn} - (1/\gamma_{l,i}) J_l^{grow} - J_{l,i}^{sink,S} \tag{12}$$

$$\frac{d[\hat{M}_{l,j}]}{dt} = J_{l,j}^{syn} + J_{l,j}^{upt,M} - J_{l,j}^{leak} - (1/\gamma_{l,j}) J_l^{grow} - J_{l,j}^{sink,M} \tag{13}$$

where $[\cdots]$ denotes molecular concentration. $S_{0,i}$ and $M_{0,j}$ are the concentrations of S_i and M_j in the chemostat feed medium respectively.

Bacterial growth occurs at much faster time scale compared to that associated with intracellular metabolic processes [3]. It is therefore reasonable to separate time scales by assuming quasi-steady-state for intracellular growth substrates $\hat{S}_{l,i}$ and metabolic building blocks $\hat{M}_{l,j}$. Solving Equation (12) and (13) with $d[\hat{S}_{l,i}]/dt = d[\hat{M}_{l,j}]/dt = 0$ yields the internal flux-balance equations

$$J_{l,i}^{upt,S} - \sum_{j=1}^{n_m} (1/\delta_{l,i,j}) J_{l,j}^{syn} - (1/\gamma_{l,i}) J_l^{grow} - J_{l,i}^{sink,S} = 0 \tag{14}$$

$$J_{l,j}^{syn} + J_{l,j}^{upt,M} - J_{l,j}^{leak} - (1/\gamma_{l,j}) J_l^{grow} - J_{l,j}^{sink,M} = 0 \tag{15}$$

Here, the uptake rate of the external substrate S_i by cell type l follows the classical Monod equation [4]

$$J_{l,i}^{upt,S} = \frac{V_{l,i}[S_i]}{K_{l,i} + [S_i]} \qquad l = 1, 2, \cdots, n_c, \;\; i = 1, 2, \cdots, n_s \tag{16}$$

where $V_{l,i}$ is the maximum uptake rate and $K_{l,i}$ is the half-maximal substrate concentration. Similarly, the uptake rate of the external building block M_j by cell type l follows a modified Monod equation by including terms that account for the potential inhibitions from substrates

$$J_{l,j}^{upt,M} = \frac{V_{l,j}[M_j]}{K_{l,j} + [M_j]} \left(\prod_{i=1}^{n_s} \frac{C_{l,i,j}}{C_{l,i,j} + [S_i]} \right) \qquad l = 1, 2, \cdots, n_c, \;\; j = 1, 2, \cdots, n_m \tag{17}$$

where $V_{l,j}$ is the maximum uptake rate, $K_{l,j}$ is the half-maximal metabolite concentration in the absence of the substrates S_i, and $C_{l,i,j}$ is the inhibition constant. We assume that substrates are the preferred sources and can repress uptake of metabolic building blocks if both types of resources are present in the environment. One well-known example is diauxic growth of *Escherichia coli* (*E. coli*) on glucose where acetate is secreted from the beginning but consumed only after glucose exhaustion [5]. This regulatory mechanism that determines hierarchical use of resources associated with the same limiting nutrient is generally termed as "catabolite repression" and has been universally found in both prokaryotic and eukaryotic microorganisms [6].

The total influxes of substrates $J_{l,i}^{upt,S}$ and metabolites $J_{l,j}^{upt,M}$ are balanced by their consumption fluxes. First, the influxes of substrates are allocated to biosynthesis of internal metabolites. The maximum fraction reserved by each substrate $\hat{S}_{l,i}$ to produce each metabolite $\hat{M}_{l,j}$ is quantified by $\phi_{l,i,j}$ ($0 \le \sum_{j=1}^{n_m} \phi_{l,i,j} \le 1$). Since the biosynthesis reaction of a metabolite generally couples multiple substrates as reactants through fixed stoichiometry ratio, its actual flux can be modeled through the Liebig's law of minimum, which states that the rate-limiting step of a reaction is determined by the reactant with the minimum ratio of its supply level relative to its stoichiometric coefficient (i.e., demand)

$$J_{l,j}^{syn} = \min_{i=1,2,\cdots,n_s} \left(\frac{\underbrace{\phi_{l,i,j} J_{l,i}^{upt,S}}_{\text{supply}}}{\underbrace{1/\delta_{l,i,j}}_{\text{demand}}} \right) \qquad l = 1,2,\cdots,n_c, \quad j = 1,2,\cdots,n_m \tag{18}$$

Similarly, we assume, for each metabolite $\hat{M}_{l,j}$, a constant fraction ($\varphi_{l,j}$) of the influx is released back to the environment

$$J_{l,j}^{leak} = \varphi_{l,j} J_{l,j}^{syn} \tag{19}$$

The validity of this assumption is discussed in Sect. 5.

Second, the remaining influxes of substrates and metabolites are supplied to biomass production. The specific growth rate of cell type l can also be modeled through the Liebig's law of minimum (i.e., rate is limited by the substrate or metabolite with the minimum supply-demand ratio)

$$J_{l}^{grow} = \underbrace{\min\left(\min_{i=1,2,\cdots,n_s} \left(\frac{\underbrace{J_{l,i}^{upt,S} - \sum_{j=1}^{n_m} (1/\delta_{l,i,j}) J_{l,j}^{syn}}_{\text{supply}}}{\underbrace{1/\gamma_{l,i}}_{\text{demand}}} \right), \min_{j=1,2,\cdots,n_m} \left(\frac{\underbrace{J_{l,j}^{syn} - J_{l,j}^{leak} + J_{l,j}^{upt,M}}_{\text{supply}}}{\underbrace{1/\gamma_{l,j}}_{\text{demand}}} \right) \right)}_{\text{basal growth rate}}$$

$$\cdot \underbrace{\left(\prod_{i=1}^{n_s} \frac{I_{l,i}}{I_{l,i} + [S_i]} \right)}_{\text{inhibition due to substrate toxicity}} \cdot \underbrace{\left(\prod_{j=1}^{n_m} \frac{I_{l,j}}{I_{l,j} + [M_j]} \right)}_{\text{inhibition due to metabolite toxicity}} \qquad l = 1,2,\cdots,n_c \tag{20}$$

where we take the inhibitory effects on growth for substrates and metabolites if they are also toxic and $I_{l,i}$, $I_{l,j}$ represent the half-inhibition concentrations of the substrate $\hat{S}_{l,i}$ and the metabolite $\hat{M}_{l,j}$ respectively.

Finally, the remaining substrate influxes that are neither converted to metabolites nor incorporated into biomass, and the remaining metabolite influxes that are neither leaked to the environment nor incorporated into biomass, are dumped through their sink reactions

$$J_{l,i}^{sink,S} = J_{l,i}^{upt,S} - \sum_{j=1}^{n_m} (1/\delta_{l,i,j}) J_{l,j}^{syn} - (1/\gamma_{l,i}) J_{l}^{grow} \tag{21}$$

$$J_{l,j}^{sink,M} = J_{l,j}^{syn} + J_{l,j}^{upt,M} - J_{l,j}^{leak} - (1/\gamma_{l,j}) J_{l}^{grow} \tag{22}$$

Since we take the min form of the fluxes involving substrates and metabolites (Equation (18) and (20)), only the most growth-limiting nutrient (either a substrate of a metabolite) that is in short supply is explicitly conserved.

The per-capita mortality rate of cell type l is assume to be a constant

$$J_l^{death} = \eta_l \qquad l = 1, 2, \cdots, n_c \tag{23}$$

2 Unilateral Cross-Feeding between Glucose and Acetate Specialists

2.1 Model Specification from the General Framework

Our first application is a community of two *E. coli* mutants (CV103 and CV101) with different strategies of resource utilization [7]. Briefly, the CV103 mutant has faster glucose uptake rate than the CV101 mutant. However, it cannot utilize acetate, while CV101 can grow on acetate and co-utilize both carbon sources. By secreting acetate, CV103 creates an additional carbon-source niche for CV101 and the two mutants are thus involved in a one-way cross-feeding interaction.

A chemostat model was derived using the framework we described in Sect. 1

$$\frac{d[G]}{dt} = D(G_0 - [G]) - J_{1,g}^{upt} N_1 - J_{3,g}^{upt} N_3 \tag{24}$$

$$\frac{dN_1}{dt} = N_1 \left(J_1^{grow} - J_1^{death} - D \right) \tag{25}$$

$$\frac{dN_3}{dt} = N_3 \left(J_3^{grow} - J_3^{death} - D \right) \tag{26}$$

$$\frac{d[A]}{dt} = D(A_0 - [A]) + \left(J_{1,a}^{leak} - J_{1,a}^{upt} \right) N_1 + J_{3,a}^{leak} N_3 \tag{27}$$

where D is the dilution rate, G_0 and A_0 are the feed medium concentrations of glucose and acetate respectively, $[G]$ and $[A]$ are their concentrations in the culture vessel respectively, N_1 and N_3 are the active population densities of CV101 and CV103 respectively.

Reaction rates (J's) are described as follows. $J_{1,g}^{upt}$ and $J_{3,g}^{upt}$ are the glucose uptake rates for the two mutants

$$J_{1,g}^{upt} = \frac{V_{1,g}[G]}{K_g + [G]} \tag{28}$$

$$J_{3,g}^{upt} = \frac{V_{3,g}[G]}{K_g + [G]} \tag{29}$$

where $V_{1,g}$ and $V_{3,g}$ are the maximum glucose uptake rates for CV101 and CV103 respectively, and K_g is the half-saturation glucose concentration (we assume the same value for both mutants). $J_{1,a}^{upt}$ is the acetate uptake rate for CV101

$$J_{1,a}^{upt} = \frac{V_{1,a}[A]}{K_{1,a} + [A]} \frac{C_{1,g}}{C_{1,g} + [G]} \tag{30}$$

where $V_{1,a}$ is the maximum uptake rate, $K_{1,a}$ is half-saturation acetate concentration in the absence of glucose, and $C_{1,g}$ is the inhibition constant. Since the acetyl-CoA synthetase was semi-constitutively expressed in CV101 [7], we assume that the glucose repression of acetate uptake is not fully relieved and the repression effect can be quantified by $C_{1,g}$.

The acetate production rates in both CV101 $\left(J_{1,a}^{syn}\right)$ and CV103 $\left(J_{3,a}^{syn}\right)$ cells are proportional to their corresponding glucose uptake rates

$$J_{1,a}^{syn} = \phi_a \delta_a J_{1,g}^{upt} \tag{31}$$

$$J_{3,a}^{syn} = \phi_a \delta_a J_{3,g}^{upt} \tag{32}$$

where ϕ_a is the fraction of glucose uptake allocated to produce acetate and δ_a is the number of acetate produced per molecule of glucose consumed (we assume both parameters share the same values between

CV101 and CV103). The acetate leakage rates by both CV101 $\left(J_{1,a}^{leak}\right)$ and CV103 $\left(J_{3,a}^{leak}\right)$ cells are then proportional to their corresponding acetate production rates

$$J_{1,a}^{leak} = \varphi_a J_{1,a}^{syn} \tag{33}$$
$$J_{3,a}^{leak} = \varphi_a J_{3,a}^{syn} \tag{34}$$

where φ_a is the proportion of acetate that is leaked to the environment (again, we assume it has the same value between CV101 and CV103).

The per-capita growth rates for CV101 $\left(J_1^{grow}\right)$ and CV103 $\left(J_3^{grow}\right)$ are determined by the most limiting nutrient supply between acetate and the remaining glucose that is not converted to acetate

$$J_1^{grow} = \min\left(\gamma_g J_{1,g}^{upt}(1-\phi_a),\ \gamma_a\left(J_{1,a}^{syn} - J_{1,a}^{leak} + J_{1,a}^{upt}\right)\right)\frac{I_{1,a}}{I_{1,a}+[A]} \tag{35}$$
$$J_3^{grow} = \min\left(\gamma_g J_{3,g}^{upt}(1-\phi_a),\ \gamma_a\left(J_{3,a}^{syn} - J_{3,a}^{leak}\right)\right)\frac{I_{3,a}}{I_{3,a}+[A]} \tag{36}$$

Note that the flux of remaining glucose is a proxy of building blocks that cannot be synthesized from acetate. Therefore, γ_a is the biomass yield of *E. coli* on acetate and γ_g represents the averaged yield value for *E. coli* cells to grow on building blocks other than acetate. $I_{1,a}$ and $I_{3,a}$ are the thresholds of growth inhibition of CV101 and CV103 by acetate respectively.

Lastly, cell death is not considered in this model so that

$$J_1^{death} = J_3^{death} = 0 \tag{37}$$

2.2 Simplifying Assumptions and Justifications

Biomass yields on glucose and acetate are similar for *E. coli* cells [8, 9]: The yield of glucose was reported to be 0.45 gDW/g glucose (equivalent to 13.5 gDW/mol Carbon) [8] and the observed per-carbon acetate yield is between 10-15 gDW/mol Carbon, depending on the acetate level [9]. It is therefore reasonable to assume that glucose and acetate are completely substitutable and all building blocks that are synthesized from glucose can also be synthesized from acetate. With this assumption, we can assume that 100% of glucose influx is directed to synthesize acetate, i.e., $\phi_a = 1$, and as a result, acetate is the only growth-limiting factor.

The schematic diagram of the simplified model is shown in Fig. 2A in the main text and its equations are described below

$$\frac{d[G]}{dt} = D(G_0 - [G]) - J_{1,g}^{upt}N_1 - J_{3,g}^{upt}N_3 \tag{38}$$
$$\frac{dN_1}{dt} = N_1\left(J_1^{grow} - D\right) \tag{39}$$
$$\frac{dN_3}{dt} = N_3\left(J_3^{grow} - D\right) \tag{40}$$
$$\frac{d[A]}{dt} = D(A_0 - [A]) + \left(\varphi_a\delta_a J_{1,g}^{upt} - J_{1,a}^{upt}\right)N_1 + \varphi_a\delta_a J_{3,g}^{upt}N_3 \tag{41}$$
$$J_{1,g}^{upt} = \frac{V_{1,g}[G]}{K_g+[G]} \tag{42}$$
$$J_{3,g}^{upt} = \frac{V_{3,g}[G]}{K_g+[G]} \tag{43}$$

$$J_{1,a}^{upt} = \frac{V_{1,a}[A]}{K_{1,a}+[A]}\frac{C_{1,g}}{C_{1,g}+[G]} \tag{44}$$

$$J_{1}^{grow} = \gamma_a\left((1-\varphi_a)\delta_a J_{1,g}^{upt} + J_{1,a}^{upt}\right)\frac{I_{1,a}}{I_{1,a}+[A]} \tag{45}$$

$$J_{3}^{grow} = \gamma_a(1-\varphi_a)\delta_a J_{3,g}^{upt}\frac{I_{3,a}}{I_{3,a}+[A]} \tag{46}$$

3 Bilateral Cross-Feeding between Lysine and Leucine auxotrophies

3.1 Model Specification from the General Framework

Our second application is a community of two *E. coli* single-gene knockout strains: knockouts of *lysA* and *leuA* genes resulted in two engineered strains that have auxotrophic phenotype of lysine and leucine respectively [10]. The two mutants cooperate by compensating for each other's metabolic deficiency: the lysine auxotroph (ΔK) secretes leucine that can be utilized by the leucine auxotroph (ΔL), which in return facilitate growth of the lysine auxotroph by secreting lysine to the environment.

A chemostat model was derived using the framework we developed in Sect. 1

$$\frac{d[G]}{dt} = D(G_0 - [G]) - J^{upt}_{\Delta k,g} N_{\Delta k} - J^{upt}_{\Delta l,g} N_{\Delta l} \tag{47}$$

$$\frac{dN_{\Delta k}}{dt} = \left(J^{grow}_{\Delta k} - J^{death}_{\Delta k} - D\right) N_{\Delta k} \tag{48}$$

$$\frac{dN_{\Delta l}}{dt} = \left(J^{grow}_{\Delta l} - J^{death}_{\Delta l} - D\right) N_{\Delta l} \tag{49}$$

$$\frac{d[K]}{dt} = D(K_0 - [K]) + J^{leak}_{\Delta l,k} N_{\Delta l} - J^{upt}_{\Delta k,k} N_{\Delta k} - J^{upt}_{\Delta l,k} N_{\Delta l} \tag{50}$$

$$\frac{d[L]}{dt} = D(L_0 - [L]) + J^{leak}_{\Delta k,l} N_{\Delta k} - J^{upt}_{\Delta k,l} N_{\Delta k} - J^{upt}_{\Delta l,l} N_{\Delta l} \tag{51}$$

where D is the dilution rate, G_0, K_0 and L_0 are the feed medium concentrations of glucose, lysine and leucine respectively, $[G]$, $[K]$ and $[L]$ are their concentrations in the culture vessel respectively, $N_{\Delta k}$ and $N_{\Delta l}$ are the population densities of active cells of the lysine and leucine auxotroph respectively.

Reaction rates (J's) are described as follows. Since the two *E. coli* auxotrophies are identical except that each carries a knockout of a single different gene, they were assumed to have equal glucose uptake kinetics, i.e.,

$$J^{upt}_{\Delta k,g} = J^{upt}_{\Delta l,g} = \frac{V_g[G]}{K_g + [G]} \tag{52}$$

where V_g is the maximum uptake rate and K_g is the half-saturation glucose concentration. $J_{\Delta k,l}$, $J_{\Delta l,k}$, $J_{\Delta k,k}$ and $J_{\Delta l,l}$ are the leucine uptake rate by the lysine auxotroph, the lysine uptake rate by the leucine auxotroph, the lysine uptake rate by the lysine auxotroph, and the leucine uptake rate of the leucine auxotroph respectively

$$J^{upt}_{\Delta k,k} = \frac{V_{\Delta k,k}[K]}{K_{\Delta k,k} + [K]} \tag{53}$$

$$J^{upt}_{\Delta l,l} = \frac{V_{\Delta l,l}[L]}{K_{\Delta l,l} + [L]} \tag{54}$$

$$J^{upt}_{\Delta l,k} = \frac{V_{\Delta l,k}[K]}{K_{\Delta l,k} + [K]} \tag{55}$$

$$J^{upt}_{\Delta k,l} = \frac{V_{\Delta k,l}[L]}{K_{\Delta k,l} + [L]} \tag{56}$$

where $V_{\Delta k,k}$, $V_{\Delta l,l}$, $V_{\Delta l,k}$, and $V_{\Delta k,l}$ are the maximum uptake rates and $K_{\Delta k,k}$, $K_{\Delta l,l}$, $K_{\Delta l,k}$ and $K_{\Delta k,l}$ are the half-saturation constants. We assume negligible inhibitory effects of glucose on amino acids uptake since no strong evidence has been found in literature.

$J^{leak}_{\Delta l,k}$ and $J^{leak}_{\Delta k,l}$ are the lysine leakage rate of the leucine auxotroph and the leucine leakage rate of the lysine auxotroph respectively

$$J^{leak}_{\Delta l,k} = \varphi_{\Delta l,k} J^{syn}_{\Delta l,k} \tag{57}$$
$$J^{leak}_{\Delta k,l} = \varphi_{\Delta k,l} J^{syn}_{\Delta k,l} \tag{58}$$

where $\varphi_{\Delta l,k}$ and $\varphi_{\Delta k,l}$ represent the proportions of lysine and leucine released back to the environment by the leucine and lysine auxotroph respectively. The biosynthesis rate of internal lysine by the leucine auxotroph $\left(J^{syn}_{\Delta l,k}\right)$ and that of internal leucine by the lysine auxotroph $\left(J^{syn}_{\Delta k,l}\right)$ are proportional to their corresponding glucose uptake rates

$$J^{syn}_{\Delta l,k} = \phi_{\Delta l,k} \delta_k J^{upt}_{\Delta l,g} \tag{59}$$
$$J^{syn}_{\Delta k,l} = \phi_{\Delta k,l} \delta_l J^{upt}_{\Delta k,g} \tag{60}$$

where $\phi_{\Delta l,k}$ and $\phi_{\Delta k,l}$ are the fractions of glucose influx allocated to produce lysine by the leucine auxotroph and leucine by the lysine auxotroph respectively, and δ_k and δ_l are the number of lysine and leucine molecules produced per molecule of glucose consumed respectively.

The per-capita growth rate of each auxotroph is determined by the most limiting factor between the auxotrophic amino acid (i.e., leucine for the leucine auxotroph and lysine for the lysine auxotroph), the non-auxotrophic amino acid (i.e., leucine for the lysine auxotroph and lysine for the leucine auxotroph), and the remaining glucose that is not converted to the non-auxotrophic amino acid

$$J^{grow}_{\Delta k} = \min\left(\gamma_g (1-\phi_{\Delta k,l}) J^{upt}_{\Delta k,g},\ \gamma_k J^{upt}_{\Delta k,k},\ \gamma_l (J^{upt}_{\Delta k,l} + J^{syn}_{\Delta k,l} - J^{leak}_{\Delta k,l})\right) \tag{61}$$
$$J^{grow}_{\Delta l} = \min\left(\gamma_g (1-\phi_{\Delta l,k}) J^{upt}_{\Delta l,g},\ \gamma_l J^{upt}_{\Delta l,l},\ \gamma_k (J^{upt}_{\Delta l,k} + J^{syn}_{\Delta l,k} - J^{leak}_{\Delta l,k})\right) \tag{62}$$

Note that the flux of remaining glucose is a proxy of building blocks other than the auxotrophic and non-auxotrophic amino acids. Therefore, γ_k and γ_l represent the biomass yields of *E. coli* on lysine and leucine respectively, and γ_g represents the averaged yield of other building blocks. Although excessive amounts (in mM range) of certain amino acids (including leucine) are toxic to *E. coli* [11], we assume that the growth inhibitory effects of lysine and leucine are negligible, given that their concentrations were observed to be in the range of sub-mM levels in monoculture experiments [10].

Lastly, cell mortality is modeled with a first-order kinetic rate expression

$$J^{death}_{\Delta k} = \eta_{\Delta k} \tag{63}$$
$$J^{death}_{\Delta l} = \eta_{\Delta l} \tag{64}$$

where $\eta_{\Delta k}$ and $\eta_{\Delta l}$ are the rate constants.

3.2 Simplifying Assumptions and Justifications

The model can be simplified by two assumptions: (1) leucine or lysine does not limit growth of the auxotrophic strain that synthesizes it *de novo* (i.e., its producing strain) and (2) environment leucine or lysine is not assimilated by its producing auxotrophic strain. We justified the first assumption by considering that the lysine biosynthesis pathway in the leucine auxotroph and the leucine biosynthesis pathway in the lysine auxotroph are unperturbed such that their biosynthetic fluxes may be still tightly regulated and coordinated with fluxes of other metabolic building blocks. It is therefore reasonable to assume that lysine and leucine are as growth-limiting as other building blocks (represented by the remaining glucose

flux that is not converted to the two amino acids) and never become the sole most growth-limiting factor in their producing strains.

Based on the first assumption, we can simplified the model by lumping the growth effect of lysine and leucine into the growth effect of glucose in their producing strains. As a result, growth of the lysine or leucine auxotroph only depends on the availability of the amino acid that it is auxotrophic for and the remaining glucose that is not converted to the auxotrophic amino acid. Since the consumption of lysine and leucine into biomass are implicitly modeled through glucose, their biosynthesis fluxes in the model should only include the proportion that is eventually released to the environment and thus equal to their leakage fluxes, i.e., $\varphi_{\Delta k,l} = \varphi_{\Delta l,k} = 1$.

The second assumption was justified by parameter sensitivity analysis of the model developed in Sect. 3.1 using Markov-Chain-Monte-Carlo algorithm. Note that the model in Sect. 3.1 does not take any of these assumptions. As shown in S7 Fig, the posterior distribution of amino acid uptake rates by their producing strains are 1-2 orders of magnitude lower than the distribution of amino acid uptake rates by their non-producing strains. Specifically, the median values of their posterior distributions are $V_{\Delta k,k} = 9.65\times 10^{-14}$, $V_{\Delta l,l} = 1.27\times 10^{-13}$, $V_{\Delta l,k} = 5.20\times 10^{-16}$, and $V_{\Delta k,l} = 1.07\times 10^{-15}$. Since $V_{\Delta l,k}$ and $V_{\Delta k,l}$ are orders of magnitude smaller than $V_{\Delta k,k}$ and $V_{\Delta l,l}$, we assume $V_{\Delta l,k} = V_{\Delta k,l} = 0$ in the simplified model.

The schematic diagram of the simplified model is shown in Fig. 3A in the main text and its equations are described below

$$\frac{d[G]}{dt} = D(G_0 - [G]) - J_g^{upt}(N_{\Delta k} + N_{\Delta l}) \tag{65}$$

$$\frac{dN_{\Delta k}}{dt} = \left(J_{\Delta k}^{grow} - \eta_{\Delta k} - D\right) N_{\Delta k} \tag{66}$$

$$\frac{dN_{\Delta l}}{dt} = \left(J_{\Delta l}^{grow} - \eta_{\Delta l} - D\right) N_{\Delta l} \tag{67}$$

$$\frac{d[K]}{dt} = D(K_0 - [K]) + \phi_{\Delta l,k}\delta_k J_g^{upt} N_{\Delta l} - J_{\Delta k,k}^{upt} N_{\Delta k} \tag{68}$$

$$\frac{d[L]}{dt} = D(L_0 - [L]) + \phi_{\Delta k,l}\delta_l J_g^{upt} N_{\Delta k} - J_{\Delta l,l}^{upt} N_{\Delta l} \tag{69}$$

$$J_g^{upt} = \frac{V_g[G]}{K_g + [G]} \tag{70}$$

$$J_{\Delta k,k}^{upt} = \frac{V_{\Delta k,k}[K]}{K_{\Delta k,k} + [K]} \tag{71}$$

$$J_{\Delta l,l}^{upt} = \frac{V_{\Delta l,l}[L]}{K_{\Delta l,l} + [L]} \tag{72}$$

$$J_{\Delta k}^{grow} = \min\left(\gamma_g(1 - \phi_{\Delta k,l}) J_g^{upt},\ \gamma_k J_{\Delta k,k}^{upt}\right) \tag{73}$$

$$J_{\Delta l}^{grow} = \min\left(\gamma_g(1 - \phi_{\Delta l,k}) J_g^{upt},\ \gamma_l J_{\Delta l,l}^{upt}\right) \tag{74}$$

4 Multilateral Cross-Feeding between 14 Amino Acid auxotrophies

4.1 Model Specification from the General Framework

The third application is a community of 14 *E. coli* amino acid knockouts [12], each of which is auxotrophic for cysteine (ΔC), phenylalanine (ΔF), glycine (ΔG), histidine (ΔH), isoleucine (ΔI), lysine (ΔK), leucine (ΔL), methionine (ΔM), proline (ΔP), arginine (ΔR), serine (ΔS), threonine (ΔT), tryptophan (ΔW), and tyrosine (ΔY). For simplicity, we assume that each of the 14 strains only uptakes the amino acid that it is auxotrophic for but has the potential to secrete all other 13 amino acids to the environment. This assumption was already justified for the community of the lysine and leucine auxotroph in Sect. 3.2 and directly applied here. By extending our 2-auxotroph model in Sect. 3.2, we presented the following 14-auxotroph community model

$$\frac{d[G]}{dt} = D(G_0 - [G]) - J_g^{upt} \sum_{x \in AA} N_{\Delta x} \tag{75}$$

$$\frac{dN_{\Delta x}}{dt} = \left(J_{\Delta x}^{grow} - \eta_{\Delta x} - D \right) N_{\Delta x} \qquad x \in AA \tag{76}$$

$$\frac{d[M_z]}{dt} = D(M_{0,z} - [M_z]) + \delta_z J_g^{upt} \left(\sum_{x \in AA} \varphi_{\Delta x,z} N_{\Delta x} \right) - J_{\Delta z,z}^{upt} N_{\Delta z} \qquad z \in AA \tag{77}$$

$$AA = \{c, f, g, h, i, k, l, m, p, r, s, t, w, y\} \tag{78}$$

where D is the dilution rate, G_0 and $M_{0,z}$ are the feed medium concentrations of glucose and amino acid z respectively, $[G]$ and $[M_z]$ are their concentrations in the culture vessel respectively, $N_{\Delta x}$ ($N_{\Delta z}$) is the population density of active cells of the auxotroph Δx (Δz), δ_z is the number of molecules of amino acid z produced per glucose consumed, $\varphi_{\Delta x,z}$ is the leakage fraction of amino acid z secreted by the auxotroph Δx, and $\eta_{\Delta x}$ is the mortality rate of the auxotroph Δx.

Reaction rates (J's) are described as follows. We assume that all auxotrophic strains have the same kinetics of glucose uptake because they are identical except for a single different gene knockout in each strain. J_g^{upt} is the glucose uptake rate for all auxotrophies

$$J_g^{upt} = \frac{V_g[G]}{K_g + [G]} \tag{79}$$

where V_g is the maximum uptake rate and K_g is the half-saturation glucose concentration. $J_{\Delta z,z}^{upt}$ is the uptake rate of amino acid z by its auxotrophic strain

$$J_{\Delta z,z}^{upt} = \frac{V_{\Delta z,z}[M_z]}{K_{\Delta z,z} + [M_z]} \tag{80}$$

where $V_{\Delta z,z}$ is the maximum uptake rate and $K_{\Delta z,z}$ is the half-saturation concentration of amino acid z. The per-capita growth rate of each auxotroph is determined by the more limiting factor between the auxotrophic amino acid and the remaining glucose that is not converted to the amino acid (proxy of building blocks other than the amino acid)

$$J_{\Delta x}^{grow} = \min\left(\gamma_g J_g^{upt} \left(1 - \sum_{z \in AA} \varphi_{\Delta x,z} \right),\ \gamma_x J_{\Delta x,x}^{upt} \right) \tag{81}$$

where γ_x and γ_g are the biomass yields of *E. coli* on amino acid x and building blocks other than amino acid x respectively.

4.2 Simplified Pairwise Batch Co-culture Model

Here we derive the analytical solution of population density fold change in pairwise coculture between any two auxotrophic strains (e.g., Δx and Δz). Given the following assumptions,

- All cells are active, i.e., $\eta_{\Delta x} = \eta_{\Delta z} = 0$;
- Dynamics of amino acids reach equilibrium very fast, i.e., $d[M_x]/dt = d[M_z]/dt = 0$;
- Growth of both auxotrophies are limited by the auxotrophic amino acids, i.e., $J_{\Delta x}^{grow} = \gamma_x J_{\Delta x,x}^{upt}$, $J_{\Delta z}^{grow} = \gamma_z J_{\Delta z,z}^{upt}$.

, Equations (75)-(77) can be simplified to

$$\frac{d[G]}{dt} = -J_g^{upt}(N_{\Delta x} + N_{\Delta z}) \tag{82}$$

$$\frac{dN_{\Delta x}}{dt} = \gamma_x \varphi_{\Delta z,x} \delta_x J_g^{upt} N_{\Delta z} \tag{83}$$

$$\frac{dN_{\Delta z}}{dt} = \gamma_z \varphi_{\Delta x,z} \delta_z J_g^{upt} N_{\Delta x} \tag{84}$$

, and further rewritten as

$$G(0) + \frac{N_{\Delta x}(0)}{\gamma_x \varphi_{\Delta z,x} \delta_x} + \frac{N_{\Delta z}(0)}{\gamma_z \varphi_{\Delta x,z} \delta_z} = G(t) + \frac{N_{\Delta x}(t)}{\gamma_x \varphi_{\Delta z,x} \delta_x} + \frac{N_{\Delta z}(t)}{\gamma_z \varphi_{\Delta x,z} \delta_z} \tag{85}$$

$$\frac{N_{\Delta x}(0)^2}{\gamma_x \varphi_{\Delta z,x} \delta_x} - \frac{N_{\Delta z}(0)^2}{\gamma_z \varphi_{\Delta x,z} \delta_z} = \frac{N_{\Delta x}(t)^2}{\gamma_x \varphi_{\Delta z,x} \delta_x} - \frac{N_{\Delta z}(t)^2}{\gamma_z \varphi_{\Delta x,z} \delta_z} \tag{86}$$

$G(0)$ and $G(t)$ are the glucose concentration at time 0 and time t respectively. $N_{\Delta x}(0)$ ($N_{\Delta z}(0)$) and $N_{\Delta x}(t)$ ($N_{\Delta z}(t)$) are the population density of the auxotroph Δx (Δz) at time 0 and time t respectively. At any moment t, the population densities of the two auxotrophies are given by

$$N_{\Delta x}(t) = N_{\Delta x}(0) + \frac{-\Delta_1 + \sqrt{\Delta_1^2 + \Delta_2 \Delta_3}}{\Delta_2} \gamma_x \varphi_{\Delta z,x} \delta_x \tag{87}$$

$$N_{\Delta z}(t) = N_{\Delta z}(0) + \left(G(0) - G(t) + \frac{N_{\Delta x}(0) - N_{\Delta x}(t)}{\gamma_x \varphi_{\Delta z,x} \delta_x} \right) \gamma_z \varphi_{\Delta x,z} \delta_z \tag{88}$$

where the Δ's are defined as

$$\Delta_1 = N_{\Delta x}(0) + N_{\Delta z}(0) + (G(0) - G(t)) \gamma_z \varphi_{\Delta x,z} \delta_z \tag{89}$$

$$\Delta_2 = \gamma_x \varphi_{\Delta z,x} \delta_x - \gamma_z \varphi_{\Delta x,z} \delta_z \tag{90}$$

$$\Delta_3 = \left((G(0) - G(t)) \gamma_z \varphi_{\Delta x,z} \delta_z + 2N_{\Delta z}(0) \right) (G(0) - G(t)) \tag{91}$$

The fold change of cell density is calculated as (final cell density)/(initial cell density), i.e., $N_{\Delta x}(\infty)/N_{\Delta x}(0)$ for the auxotroph Δx and $N_{\Delta z}(\infty)/N_{\Delta z}(0)$ for the auxotroph Δz. Since glucose is depleted after sufficient long time, we used $G(\infty) = 0$ in these calculations.

5 Discussions on the Proportionality Assumption for Leakage Rate

In the general modeling framework described in Sect. 1, we assumed that the leakage rate of a metabolite is proportional to its influx rate (proportionality assumption). To understand when the proportionality assumption is valid and how it breaks down, we leveraged our previous experiences in modeling *E. coli* resource allocation [13, 14] and developed a coarse-grained single-strain model that explicitly considers metabolite concentration by characterizing the kinetic rates of metabolite biosynthesis, its passive leakage, and its utilization for biomass under enzymatic regulations. A schematic diagram of the model is shown in S11A Fig. We summarize the main assumptions below

- We consider three reactions in the model: biosynthesis of an internal metabolite M from extracellular substrate S, leakage of the metabolite, and consumption of the metabolite in biomass production (reaction rate J^{con}). The biosynthetic reaction is governed by enzyme E_1 and the downstream consumption of the metabolite is mediated by enzyme E_2 whose activity can be inhibited by E_2-targeting antibiotic A. Metabolite leakage is assumed to be passive or active with a constant enzyme level. The total protein density (i.e., amino acid concentration) of E_1 and E_2 is a constant α;

- The E_2 protein production rate is proportional to the translational capacity allocated to its biosynthesis, which further equals to the total protein synthesis rate allocated to E_1 and E_2 together multiplied by the relative partitioning between the two. The total production rate of E_1 and E_2 is equal to $J^{grow}\alpha$, where J^{grow} is the specific growth rate and α, as defined above, is the concentration of total amino acids contained in E_1 and E_2. This is because the specific growth rate in balanced growth is defined as $(dX/dt)/X$, i.e., the mass production rate of any biological component X per mass of X. When X represents the total number of amino acids (as an approximation of mass) in E_1 and E_2, specific growth rate is then equal to the amino acid production rate of E_1 and E_2 per total amino acids in E_1 and E_2 (i.e., α). Therefore, the amino acid production rate of E_1 and E_2 is equal to the specific growth rate J^{grow} multiplied by α;

- The relative partitioning of the translational resources between E_1 and E_2 proteins is mediated by a transcriptional regulator R, whose biosynthesis rate is assumed to be inversely proportional to the internal metabolite concentration $[M]$. Higher R concentration leads to decreased allocation of translational resources to E_2 and, concomitantly, increased allocation to E_1. The underlying logic of this regulatory architecture is that, shortage of the internal metabolite M signals bacteria to reduce its consumption flux towards biomass while increasing the flux of its uptake and conversion. For example, biosynthesis of ppGpp (guanosine pentaphosphate) in response to amino acid shortage directly inhibits transcription of both ribosomal RNAs and ribosomal protein genes while promoting that of amino acid biosynthetic genes [13].

- The internal metabolite M and proteins E_1, E_2 are stable in exponential phase and thus not actively degraded (but they are still subject to growth dilution). By contrast, the turnover rate of R is generally much faster than the dilution rate (e.g., the half-life time of ppGpp is only 20-30 s [15]). We therefore assume first-order kinetics for its active degradation and ignore the dilution effect.

These assumptions can be translated into the following differential equations

$$\frac{d[M]}{dt} = \underbrace{\frac{k_{e,1}[E_1][S]}{K_{m,s}+[S]}}_{\substack{\text{metabolite}\\ \text{biosynthesis rate}}} - \underbrace{J^{con}}_{\substack{\text{metabolite}\\ \text{consumption rate}}} - \underbrace{J^{grow}[M]}_{\substack{\text{metabolite}\\ \text{dilution rate}}} - \underbrace{k_m([M]-M_e)}_{\substack{\text{metabolite}\\ \text{leakage rate}}} \tag{92}$$

$$\frac{d[E_2]}{dt} = \underbrace{\frac{\alpha J^{grow}}{m_{e,2}}}_{\substack{\text{maximum translational}\\ \text{capacity of } E_2}} \cdot \underbrace{\frac{K_{i,r}}{K_{i,r}+[R]}}_{\text{transcriptional regulation}} - \underbrace{J^{grow}[E_2]}_{\substack{E_2\\ \text{dilution rate}}} \tag{93}$$

$$\frac{d[R]}{dt} = \underbrace{\frac{k_r K_{m,m}}{K_{m,m}+[M]}}_{\substack{\text{transcriptional regulator}\\ \text{biosynthesis rate}}} - \underbrace{d_r[R]}_{\substack{\text{transcriptional regulator}\\ \text{degradation rate}}} \tag{94}$$

$$J^{con} = \underbrace{\frac{k_{e,2}[E_2][M]}{K_{m,m}+[M]}}_{\substack{\text{maximum metabolite}\\ \text{consumption rate}}} \cdot \underbrace{\frac{K_{i,a}}{K_{i,a}+[A]}}_{\text{antibiotic inhibition}} \tag{95}$$

$$J^{grow} = \underbrace{J^{con}}_{\substack{\text{metabolite}\\ \text{consumption rate}}} \cdot \underbrace{Y_m}_{\substack{\text{yield of}\\ \text{metabolite}}} \tag{96}$$

$$[E_1] = \underbrace{\frac{\alpha - m_{e,2}[E_2]}{m_{e,1}}}_{\substack{\text{conservation of amino acids}\\ \text{in } E_1 \text{ and } E_2}} \tag{97}$$

where $k_{e,1}$ is the maximum metabolite biosynthesis rate from substrate (depends on nutrient quality of substrate), $k_{e,2}$ is the maximum rate of metabolite consumption, $K_{m,s}$ and $K_{m,m}$ are the Michaelis constants, k_m is the diffusion rate constant, M_e is the concentration of metabolite in the environment, α is the total amino acids contained in protein E_1 and E_2, $K_{i,r}$ is the half-inhibition constant, k_r is the maximum biosynthesis rate of the transcriptional regulator, d_r is the first-order rate constant of its degradation, $m_{e,1}$ and $m_{e,2}$ are the number of amino acids in protein E_1 and E_2 respectively, and Y_m is the yield of bacterial growth on the internal metabolite. The proportionality constant (φ) (i.e., flux ratio) between the metabolite leakage flux and its total influx is defined as

$$\varphi = \frac{k_m([M]-M_e)}{\frac{k_{e,1}[E_1][S]}{K_{m,s}+[S]}} \tag{98}$$

We consider a well-known example where S represents glucose, M represents amino acids, E_1 represents metabolic enzymes, E_2 represents ribosomes (ribosomal proteins), and R represents ppGpp. Using parameter values listed in S4 Table, we simulated the steady state responses of the proportionality constant φ under two types of perturbations: (1) changing the external substrate concentration $[S]$ (S11B-E Fig) and (2) changing the external antibiotic concentration $[A]$ (S11F-I Fig). We also varied the diffusion rate constant k_m in the simulations. Our simulation results suggest the following

- Increasing external substrate concentration increases growth rate (S11B Fig) while increasing antibiotic concentration decreases growth rate (S11F Fig);
- Increasing external substrate concentration leads to a positive correlation between the influx of the internal metabolite and its concentration (S11C Fig) while increasing antibiotic concentration leads to a negative correlation (S11G Fig);
- Increasing external substrate concentration and antibiotic concentration both lead to higher concentration of the internal metabolite (S11D,H Fig) ;

- φ remains unchanged by increasing external substrate concentration (S11E Fig) but it increases substantially at higher antibiotic concentration (S11I Fig).

From the findings above, we conclude that the proportionality assumption may be valid for an internal metabolite when its concentration is perturbed from the upstream of the metabolite (e.g., change external substrate concentration) since the assumption couples the leakage with upstream biosynthesis. However, it is shown to break down when the perturbation is applied from the downstream of the metabolite (e.g., change ribosome-targeting antibiotic concentration). This is also expected because the assumption does not take feedback regulation from the downstream reactions and metabolites into accounts.

6 References

[1] David Tilman. *Resource competition and community structure*. Princeton university press, 1982.

[2] Robert Marsland III, Wenping Cui, Joshua Goldford, Alvaro Sanchez, Kirill Korolev, and Pankaj Mehta. Available energy fluxes drive a transition in the diversity, stability, and functional structure of microbial communities. *PLoS computational biology*, 15(2):e1006793, 2019.

[3] Thibaud Taillefumier, Anna Posfai, Yigal Meir, and Ned S Wingreen. Microbial consortia at steady supply. *Elife*, 6:e22644, 2017.

[4] Jacques Monod. The growth of bacterial cultures. *Annual Review of Microbiology*, 3(1):371–394, 1949.

[5] William F Loomis and Boris Magasanik. Glucose-lactose diauxie in *Escherichia coli*. *Journal of bacteriology*, 93(4):1397–1401, 1967.

[6] Jr MH Saier. A multiplicity of potential carbon catabolite repression mechanisms in prokaryotic and eukaryotic microorganisms. *The New Biologist*, 3(12):1137–1147, 1991.

[7] R Frank Rosenzweig, RR Sharp, David S Treves, and Julian Adams. Microbial evolution in a simple unstructured environment: genetic differentiation in *Escherichia coli*. *Genetics*, 137(4):903–917, 1994.

[8] Hannes Link, Bernd Anselment, and Dirk Weuster-Botz. Leakage of adenylates during cold methanol/glycerol quenching of *Escherichia coli*. *Metabolomics*, 4(3):240–247, 2008.

[9] Brice Enjalbert, Pierre Millard, Mickael Dinclaux, Jean-Charles Portais, and Fabien Létisse. Acetate fluxes in *Escherichia coli* are determined by the thermodynamic control of the Pta-AckA pathway. *Scientific Reports*, 7:42135, 2017.

[10] Xiaolin Zhang and Jennifer L Reed. Adaptive evolution of synthetic cooperating communities improves growth performance. *PLoS ONE*, 9(10):e108297, 2014.

[11] Irem Avcilar-Kucukgoze, Alexander Bartholomäus, Juan A Cordero Varela, Robert Franz-Xaver Kaml, Peter Neubauer, Nediljko Budisa, and Zoya Ignatova. Discharging tRNAs: a tug of war between translation and detoxification in *Escherichia coli*. *Nucleic Acids Research*, 44(17):8324–8334, 2016.

[12] Michael T Mee, James J Collins, George M Church, and Harris H Wang. Syntrophic exchange in synthetic microbial communities. *Proceedings of the National Academy of Sciences*, 111(20):E2149–E2156, 2014.

[13] Chen Liao, Andrew E Blanchard, and Ting Lu. An integrative circuit–host modelling framework for predicting synthetic gene network behaviours. *Nature microbiology*, 2(12):1658–1666, 2017.

[14] Chen Liao. *Integrated modeling of bacterial metabolism*. PhD thesis, University of Illinois at Urbana-Champaign, 2018.

[15] R Voellmy and AL Goldberg. Guanosine-5'-diphosphate-3'-diphosphate (ppgpp) and the regulation of protein breakdown in *Escherichia coli*. *J Biol Chem*, 255(3):1008–1014, 1980.

[16] Ivana Gudelj, Margie Kinnersley, Peter Rashkov, Karen Schmidt, and Frank Rosenzweig. Stability of cross-feeding polymorphisms in microbial communities. *PLoS computational biology*, 12(12):e1005269, 2016.

[17] Emmanuel Anane, Peter Neubauer, M Nicolas Cruz Bournazou, et al. Modelling overflow metabolism in *Escherichia coli* by acetate cycling. *Biochemical Engineering Journal*, 125:23–30, 2017.

[18] Rishi Jain and Ranjan Srivastava. Metabolic investigation of host/pathogen interaction using MS2-infected *Escherichia coli*. *BMC Systems Biology*, 3(1):121, 2009.

[19] Arijit Maitra and Ken A Dill. Bacterial growth laws reflect the evolutionary importance of energy efficiency. *Proc. Natl. Acad. Sci. USA*, 112(2):406–411, 2015.

[20] Yeheskel S Halpern. Genetics of amino acid transport in bacteria. *Annual Review of Genetics*, 8(1):103–133, 1974.

[21] Jeanette R Piperno and Dale L Oxender. Amino acid transport systems in *Escherichia coli* K12. *Journal of Biological Chemistry*, 243(22):5914–5920, 1968.

[22] Joseph Shiloach and Rephael Fass. Growing *E. coli* to high cell density–a historical perspective on method development. *Biotechnology Advances*, 23(5):345–357, 2005.

[23] Bonnie A Templeton and Michael A Savageau. Transport of biosynthetic intermediates: homoserine and threonine uptake in *Escherichia coli*. *Journal of bacteriology*, 117(3):1002–1009, 1974.

[24] Iwao Ohtsu, Yusuke Kawano, Marina Suzuki, Susumu Morigasaki, Kyohei Saiki, Shunsuke Yamazaki, Gen Nonaka, and Hiroshi Takagi. Uptake of L-cystine via an ABC transporter contributes defense of oxidative stress in the L-cystine export-dependent manner in *Escherichia coli*. *PLoS ONE*, 10(4):e0120619, 2015.

[25] Robert Landick, Dale L Oxender, and Giovanna Ferro-Luzzi Ames. Bacterial amino acid transport systems. In *The Enzymes of Biological Membranes*, pages 577–615. Springer, 1985.

[26] Sharon D Cosloy. D-serine transport system in *Escherichia coli* k-12. *Journal of bacteriology*, 114(2):679–684, 1973.

[27] Matthias Quick and Jonathan A Javitch. Monitoring the function of membrane transport proteins in detergent-solubilized form. *Proceedings of the National Academy of Sciences*, 104(9):3603–3608, 2007.

[28] Arvind Natarajan and Friedrich Srienc. Dynamics of glucose uptake by single *Escherichia coli* cells. *Metabolic Engineering*, 1(4):320–333, 1999.

[29] Allen G Marr. Growth rate of *Escherichia coli*. *Microbiol. Rev.*, 55(2):316–333, 1991.

[30] Stefan Klumpp, Matthew Scott, Steen Pedersen, and Terence Hwa. Molecular crowding limits translation and cell growth. *Proc. Natl. Acad. Sci. USA*, 110(42):16754–16759, 2013.
