# Supplementary material for: Modeling microbial cross-feeding at intermediate scale portrays community dynamics and species coexistence
Source: PLoS Comput Biol. 2020 Aug 18;16(8):e1008135. doi: 10.1371/journal.pcbi.1008135 (PMC7480867; doi:10.1371/journal.pcbi.1008135)
Supplement: Attachment — Submitted filename: Response_to_review_comments_final.pdf [file pcbi.1008135.s017.pdf]

Editor Comments:

Comment 1: "*Both the reviewer expressed interest in the finding of the paper, but also raised a few concerns regarding the model, the presentation of the results, and the parameter fitting. Also, please note that, as written in the instructions, stating "data available on request from the author" is not sufficient.*"

Author Reply: We would like to thank you and the two anonymous referees for taking time to read our manuscript and provide constructive feedback: We are pleased that they liked our paper. We have addressed the key concerns and revised the manuscript based on the comments of the two referees. We also make sure that all data are available within the manuscript or its supplementary materials. Please see below for our detailed point-to-point responses.

Reply to Referee #1

Comment 1: "*In this manuscript, the authors used resource competition models coupled with abstract cellular metabolism to investigate microbial ecology, focusing on the problem of cross-feeding. The model is of three layers: growth substrates, metabolic intermediates, and biomass. Variants of the model with different assumptions are applied to three experimental E. coli communities: acetate-mediated cross-feeding, bilateral amino-acid-mediated cross-feeding, and multilateral amino-acid-mediated cross-feeding. the experimental data are used to determine the parameters of each model, manually or by combining automatic estimation and manual calibration. Predictions about how external conditions such as resource supply, leakage fraction, antibiotics, and cheaters influence the community behavior are provided by model prediction. Overall, this manuscript is inspiring in successfully connecting a simple theory with experiments. However, I have a number of concerns regarding both the model and the presentation of results.*"

Author Reply: We thank the referee for carefully reading our manuscript and summarizing the key points. We are pleased the referee agrees with us on the interest of the study and thought that our paper is inspirational.

Comment 2a: "*(1). Leakage. My largest concern is about the flux of the metabolic intermediates (M in their model). For models of cross-feeding, conversion of intracellular substrate (S^) into intracellular intermediates (M^), then release of some of the intracellular intermediates back into the environment is crucial. However, for simplicity, in the current model no intracellular reactions are explicitly modeled (Equation S7-S9). The extracellular S is directly transformed by cells into the extracellular M, without intracellular metabolic details. Thus in the model there are two divergent pathways, one converting S into M directly in the environment, the other converting S into intracellular M^ that contributes to biomass accumulation.*"

Author Reply: The referee is correct that we did not explicitly model metabolite leakage as a reaction that diffuses intracellular metabolite to the environment. Instead, we assumed a direct conversion from substrate to extracellular metabolite without producing intracellular metabolite first. In the revised manuscript, we corrected this unrealistic feature by introducing two new

reactions that describe production of intracellular metabolites from substrates (Supplementary Equation (3)) and transport of metabolites from intracellular to extracellular space (Supplementary Equation (4)).

Comment 2b: "*Why does the model assume leakage is proportional to conversion rate from substrate to metabolite, rather than proportional to intracellular metabolite concentration? This biologically unrealistic feature of the model could cause unwanted outcomes. For example, accumulation of M^ inside a cell should presumably increase the leakage of M^ into the environment, but that cannot happen here because there is no such reaction from M^ to M. The current model could lead to strange behavior, e.g. high leakage even at very low internal metabolite concentration, or vice versa. In particular, I would expect that leakage could then depend on growth rate, i.e. a cell growing slowly because of lack of one metabolite could build up another metabolite, and therefore leak the latter at a higher rate. The current model assumes the opposite - slow growth means slow substrate uptake, which means slow leakage. The study would be much more convincing if the model treated leakage more realistically, i.e. as emerging from an internal metabolite pool rather than as a fixed fraction of intake flux. Short of that, the authors need to do much more to justify their approximation, e.g. showing for simple single-strain model that their approximation does a reasonable job compared to a more realistic model over a range of external conditions.*"

Author Reply: We thank the referee for pointing out the limitation of our modeling strategy. We agree that it would be ideal to model leakage rate as a function of metabolite concentration. However, this means that we need to model the kinetic rate of any reaction involving the metabolite as a function of its own substrates (and possibly products if feedback inhibition plays a role) to keep a consistent formalism and, more importantly, to correctly determine the metabolite concentration. In this paper, we did not consider such a full kinetic modeling scheme for the following reasons:

(1) **Simplicity of the model: Modeling dependence of reaction rate on metabolite concentration explicitly would increase the number of parameters needed to fit from experimental data.** Parameter estimation can become very complex for a large community model with many species. It is particularly difficult to do the fitting manually. Since most

cross-feeding experiments did not report intracellular metabolic fluxes and metabolite concentrations, these additional parameters would not be well constrained and hence have large uncertainties.

(2) **Focus of the study: It was not our goal to develop a theoretically comprehensive model**. Although a detailed kinetic model would be more theoretically complete, it does not necessarily help better explain the experimental data presented in the case studies. In fact, we show in our manuscript that our model that is based on a simple leakage assumption suits the data from three different cross-feeding experiments well.

(3) **Precedent in the literature: The assumption of proportionality between leakage and biosynthesis of metabolites is not uncommon in the literature**. Our model formalism was extended from the classical Resource-Consumer model (Tilman, 1982) and its many variants (Goldford et al., 2018; Marsland et al., 2019). For example, (Marsland et al., 2019) used a resource transformation matrix to specify the fraction of leaked energy from one resource in form of another.

To better understand the assumption and its consequences, we followed the referee's suggestion and we used a single-strain model to explore when the proportionality assumption remains valid and how it breaks down. We leveraged our previous experiences in modeling *Escherichia coli* growth (Liao, 2018; Liao et al., 2017) and developed a single-strain model, as the reviewer suggested, by incorporating the explicit dependence of metabolite leakage on its concentration (Supplementary Texts 1.5, Supplementary Fig. 11A). With the model, we altered two types of external conditions: (1) the substrate concentration (which affects metabolite biosynthesis) and (2) the concentration of a ribosome-targeting antibiotic (which affects metabolite consumption). The new simulation results show that the fraction of metabolite leakage remains constant by varying external substrate concentration (Supplementary Fig. 11B-E); however, it increases considerably with antibiotic concentration (Supplementary Fig. 11F-I). These results suggest that the proportionality assumption may remain valid when a metabolite is perturbed from its upstream side since the assumption couples the leakage with upstream biosynthesis. However, it break downs when the perturbation is applied from the downstream side since the assumption does not take feedback regulation from downstream reactions and metabolites into accounts.

The above results explain when our model can generate the "unwanted behavior" as the referee pointed out. For example, the referee noted that our model may lead to high leakage even at very low internal metabolite concentration. Our model could generate such behavior when the metabolite intake flux and its concentration are negatively correlated, as exemplified when the single-strain model is perturbed with higher antibiotic concentration (Supplementary Fig. 11G). Nonetheless, our model works just as expected when flux is positively correlated with concentration, as exemplified when the single-strain model is perturbed with higher substrate concentration (Supplementary Fig. 11C).

The referee also expected that metabolite leakage should depend on growth rate and occur at higher rate when its incorporation into biomass is inhibited. Indeed, we observed higher metabolite concentration (Supplementary Fig. 11H) and its leakage flux as antibiotic concentration increases and we agree with the referee that a linear relationship between intake and leakage flux is too simple for this type of perturbations.

In conclusion, the proportionality assumption remains valid in the context of this paper because resource level is the only varying external condition. But we now also acknowledge that this assumption is a limitation that may prevent us from generalizing the model to other perturbation types. We added a paragraph (lines 464-480) to discuss this particular model limitation. We appreciate the suggestion of the referee to model internal metabolite concentration and its kinetics, which helped us think more about the possible future directions, e.g., integrating the single-strain model with the current cross-feeding modeling framework to correct for the proportionality assumption.

Comment 3: " *(2) Uptake. Another seemingly unrealistic assumption concerns external amino acid uptake: "Here we assume that each auxotroph only uptakes the amino acid it is unable to produce and does not uptake other amino acids that it can synthesize. (SI p.12)" Certainly, E. coli cells in rich media that includes amino acids grow faster than in minimal media. The authors either need to justify this assumption, which seems contrary to evidence, or do a more careful job explaining how this assumption influences their results, and when it might be important to treat such uptake of external amino acids by non-auxotrophs more realistically.*"

Author Reply: The referee raises an interesting point. First, the assumption mentioned is not part of the general modeling framework, but a simplification applied to the specific example of cross-feeding among amino acid auxotrophic strains.

Then, the referee points out that "*E. coli cells in rich media that includes amino acids grow faster than in minimal media.*", which is correct for wild-type *E. coli* cells. However, we have no data showing that this is also true for amino acid auxotrophies, i.e., no support that supplementing an amino acid auxotroph with amino acids that it is capable of synthesizing accelerates their growth. A lysine auxotroph will not grow faster by supplementing another amino acid, such as leucine, if the uptake of lysine is its growth limiting step. To further justify this assumption, we built a reference model for the community of the lysine and leucine auxotroph, which is the same to the simplified model but includes all possible amino acid uptake reactions (Supplementary Fig. 6, Supplementary Texts 1.3.1). We estimated the maximum rates of these uptake reactions using Markov-Chain-Monte-Carlo algorithm (see Materials and methods in the main text for details) under the constraint of experimental data. As shown in Supplementary Fig. 7 (red box), the maximum uptake rate of amino acids by their producing strains (i.e., lysine uptake by the leucine auxotroph and leucine uptake by the lysine auxotroph) are 1-2 orders of magnitude lower than the maximum uptake rate of amino acids by their non-producing-strains (i.e., lysine by the lysine auxotroph and leucine by the leucine auxotroph), suggesting that the majority of amino acids in the environment are assimilated by their auxotrophies.

We believe this parameter sensitivity analysis provides a reasonable justification for considering the uptake of the amino acid that the strain is auxotrophic for to be its growth limiting process. The model with such assumption fits experimental data well in the two-species case (Fig. 3B,C) as well as in the community of 14 amino acid auxotrophies.

We now provide these justifications and the parameter sensitivity analysis in both main text (lines 221-226) and Supplementary Information (Supplementary Texts 1.3.2). We also present what we believe to be a fair statement that this assumption is specific to nutrient auxotrophic strains and may not be generalized to non-auxotrophic, wild-type cells (lines 226-229).

Comment 4: "*(3) Stoichiometry. In the model, the flux of intermediates follows both flux balance and stoichiometry, but flux balance and stoichiometry generally cannot be satisfied simultaneously without metabolic overflow. In the model, the flux from the environmental substrate S splits into*

three fluxes: F1: this flux is converted into environmental metabolites M; F2: this flux is converted into cellular metabolites M^ (then into biomass), and F3: whatever flux is left goes directly into biomass. However, if the growth rate takes the min form of the S and M fluxes, which implies that biomass requires a fixed stoichiometry of S and M, there is flux either from S or M (whichever is not limiting) that cannot enter biomass, and this excess flux need to overflow back into the medium."

Author Reply: The referee is correct that the min for of S and M implies that biomass requires a fixed stoichiometry of S, which raises the question of what happens with the excess flux of either S or M. Our model assumed that excess flux that is neither leaked nor incorporated into biomass, but instead is consumed in non-growth metabolic processes or through degradation. This means that only the growth-limiting nutrient is explicitly conserved in our model.

We agree that this point was left unclear in the previous version. We now added the above clarifications in Supplementary Texts 1.1. We also explicitly introduced the two additional sink reactions (Supplementary Equation (7) and (8)) to balance the excess fluxes associated with substrates and metabolites. These sinks combine all reactions that consume the substrates or metabolites but are not explicitly modeled. This change does not alter the model equations in any way, but it makes our modeling framework conceptually more complete.

Comment 5a: "*(4) Coexistence criteria. The conditions for coexistence are rather underdeveloped. First, in the SI section on coexistence criteria the authors only considered the fixed point solution (setting growth rate of all species equal to the dilution rate), but did not consider the stability of these fixed points. It is unclear whether these analytically derived (approximate) conditions are tested by simulation (and whether the phase diagrams in Figures 3 and 4 are generated from these analytical results or via simulations).*"

Author Reply: We agree that our previous version was unclear about how we determined species coexistence and their stability in the phase diagrams. The phase diagrams were all computed through simulations (integral of dynamical system over time until steady state); therefore, the coexistence regions are indeed stable. We emphasized that the phases diagrams were obtained "via simulations" in line 185 and 251 in the revised manuscript.

The referee is correct that the coexistence criteria we analytically derived in the Supplementary Information only considered the feasible equilibrium solutions instead of stable solutions. We did try to solve the stability condition but it is cumbersome and yields no insightful results, so we left it out of the manuscript.

Comment 5b: "*Second, more generally the work would benefit from clearer statements about the conditions for coexistence and possible types of coexisting metabolisms. The current model is very similar to that of Taillefumier et al. eLife 2017, which came to definite conclusions about what types of coexistence are possible and when. The current work could benefit from more definite statements along these lines, and a discussion of the relation to this previous study. Here are specific examples that would benefit from more clear statements: on ll. 205-7 "...the glucose specialist releases more acetate than the amount needed to help the acetate specialist overcome its basal growth disadvantage, causing a declining self-balancing capacity of population dynamics and reduced likelihood of coexistence." It's not clear what "likelihood" implies here. Similarly, on ll. 216-21 "Interestingly, growth of the dominant and rare auxotrophs are always limited by its auxotrophic amino acid and glucose respectively, which suggests an implicit negative feedback loop that maintains their relative abundance ratio before and after the switch: increasing population size of the dominant auxotroph impairs the growth of the rare auxotroph by consuming more glucose but eventually, its own growth is inhibited because a smaller amount of amino acid it needs to grow can be produced by its partner." This phrasing is rather complex, and to my mind ambiguous about the origins of the observed behavior. One suggestion is for the authors to produce a graphical representation of the conditions for coexistence, e.g. showing contours of growth rate = dilution rate for each species in the space of nutrient concentrations, and considering possible points where these could overlap to yield coexistence. Such a graphical approach is standard in the field and can provide clear intuition for the classes of outcomes possible.*"

Author Reply: We agree with the referee that our model is similar to (Taillefumier et al., 2017)—a paper which was inspirational in its conclusion that population dynamics controls resource pool availability to optimize metabolic strategies of different cell types at coexistence. Our study supports these findings for the coexistence of the leucine and lysine auxotroph and the allowable

nutrient utilization strategies are also shaped by resource pool availability through population dynamics (Fig. 3D,E). We now cite this paper and discuss its specific relations to our study in the Discussion section (lines 446-449).

We also rewrote almost the entire sections about the results of phase diagram and coexistence for both 2-strain communities (lines 181-211 for acetate-mediated cross-feeding and lines 249-275 for amino-acid-mediated cross-feeding). We clarified those ambiguous statements that the reviewer mentioned as specific examples by providing sufficient details on the statements without using undefined terms (e.g., likelihood). We also provided schematic illustrations to facilitate understanding. For example, we drew Fig. 3E to illustrate how leakage affects the metabolic strategies adopted by the lysine and leucine auxotroph at coexistence. Besides, we found it particular helpful to interpret the coexistence regime of the acetate-mediated cross-feeding system using the language from Chesson's coexistence theory (Barabás et al., 2018), by discussing how the fitness difference and niche difference covary with changes in acetate leakage fraction.

Finally, we followed the referee's suggestion to add contour plots of fixed-point solution in the nutritional space (Fig. 2F, Fig. 4C), which is very helpful as well. We hope these efforts can help clarify the main points and improve the readability.

Comment 6: **"***(5) Comparison to Lotka-Volterra (LV). Does the model actually perform better than an LV model? One of the central claims of the paper is that the level of modeling including explicit nutrients is superior to higher-level models. This sounds reasonable, but isn't satisfactorily demonstrated. The Authors should formulate an LV model with comparable complexity and demonstrate that the current model is actually superior. The authors do provide a comparison to the model in Mee et al., but that model is not sufficiently well explained or justified to judge whether this is a fair comparison.*"

Author Reply: We see that we had left the wrong impression that we wanted to state that a nutrient-explicit model is superior to a Lotka-Volterra. We believe this was already nicely demonstrated in (Momeni et al., 2017). In the abstract (lines 18-19), significance statement (lines 36) and introduction (line 67), we changed "the best level", "the most appropriate level", and "the appropriate level" to "an appropriate level" to avoid implying that an intermediate level of modeling is the best. In other places where we discuss the general limitation of these high-level

models (lines 59-60, 415-417), we cited (Momeni et al., 2017) to support our statements. We made sure that our paper does not make explicit claims about such superiority over models that work at other levels of detail.

Although that's not explicitly claimed, we did provide a comparison with Lotka-Volterra (LV)-type model for pairwise coculture data in Fig. 5B (Fig., 5A, B of the original manuscript). The LV-type model we chose was utilized in at least two papers (Hoek et al., 2016; Mee et al., 2014) and its equations is given by Equation (4) and (5) in the revised manuscript. The model has a similar complexity with ours in terms of the number of free parameters (198 for the LV-type model and 205 for ours). In Fig. 5B, we show that our model has a much better fit to pairwise coculture data (Pearson correlation coefficient: 94% vs. 32%), where the parameters of the LV-type model was chosen to be the sample that gives the highest Pearson correlation coefficient among 100,000 samples from Markov-Chain-Monte-Carlo simulated samples (see Materials and methods in the main text for details). We added these new results and discussions in the revised manuscript (lines 333-342).

Comment 7: "*(6) Parameter sensitivity analysis. Since the analytical results require approximations (S34-35), and the parameters are manually tuned, I think a parameter sensitivity analysis for coexistence is quite important.*"

Author Reply: As we mentioned in our response to **Comment #5a**, the coexistence region in the phase diagram (Fig. 3A of the original manuscript, Fig. 2G of the revised manuscript) was determined via simulations, rather than analytical approximation. To examine how sensitive the coexistence region is to changes in parameters, we first determined the uncertainty associated with free parameters of the model by sampling the posterior distributions using Markov-Chain-Monte-Carlo algorithm (see Materials and methods in the main text for details). We found that $C_{1,g}$ (half-inhibition constant for acetate uptake by glucose) and $I_{3,a}$ (half-inhibition constant for CV103 growth due to acetate toxicity) have the largest uncertainty on the log scale (Supplementary Fig. 3A). We then varied the two parameters around their default values used in the simulations, showing that the coexistence region remains similar in shape and position within the phase diagram (Supplementary Fig. 4). We indicated that the coexistence region is robust to change in the two most uncertain parameters in lines 192-194 of the revised manuscript.

Comment 8: "*(7) Why does the coexistence regime in Figure 3 so narrow? In Lenski's expeirment, the coexistence is quite stable.*"

Author Reply: We believe that the reviewer refers to (Rozen and Lenski, 2000) as the "Lenski's experiment". Indeed, the L and S clones isolated by (Rozen and Lenski, 2000) coexist after almost 20,000 generations. However, the strains (CV101 and CV103) and data we modeled in the manuscript are from an earlier paper by different authors (Rosenzweig et al., 1994). Even though the L/S and CV101/CV103 clones share similar phenotypic characteristics, they were evolved independently from two studies with different experimental setups (the differences were noticed and discussed in their paper (Rozen and Lenski, 2000)). It therefore remains unknown whether the community of CV101 and CV103 has similar long-term stability as the community of L/S clones in Lenski's experiment.

In addition, the experimental data from (Rosenzweig et al., 1994) shows that coculture of CV101 and CV103 remains stable over 40 generations. It is also unknown whether the coexistence state can be maintained over longer time scales (more generations). In fact, our model, which provides a great fit to the 40-generation data, predicts that CV103 would be eventually excluded from the community in the long run (Supplementary Fig. 2). The experimental data obtained within finite time is therefore not so much informative as to determine whether the coexistence regime (Fig. 3A of the original manuscript, Fig. 2G of the revised manuscript), constructed at steady state when time goes to infinity, should be narrow or wide. We remind the readers of this fundamental difference between the steady-state feature associated with the phase diagram and the transient dynamics shown by experimental data in the Results (lines 194-196) of the revised manuscript.

Comment 9: "*(8) What is the definition of a generation? Presumably ~ 1/D.*"

Author Reply: Correct. It is log(2)/D. This is consistent with the definition used in the original paper of experiment (Rosenzweig et al., 1994): *"Cells were grown in aerated culture vessels 145-210 ml in volume at a dilution rate of D = 0.2 hr, equivalent to a generation time of approximately 3.5 hr"*. We clarified its definition in the caption of Fig. 2 (line 741) in the revised manuscript.

Comment 10: "*(9) Fig. 3B - why is the leakage rate set so high, at 0.5?*"

Author Reply: The phase diagram (Fig. 3B of the original manuscript) shows the results from models where we varied the feed medium glucose concentration (x axis) and lysine leakage fraction (y axis). We kept the other parameters such as the leucine leakage fraction constant. We chose a value of 0.5 because it made coexistence possible in the phase diagram, but this result is not unique: qualitatively similar results can be achieved with leakage fractions as low as > 0.1.

In the revised manuscript, we replaced this figure with Fig. 3D that shows the phase diagram in an alternative space spanned by the two amino acid leakage fractions on the x and y axis (10mM glucose). We found this way a better presentation since we no longer need to choose an arbitrary value for one of the two leakage parameters. Moreover, the new phase space shows that coexistence requires a negative interaction between the minimum of the two leakage fractions, which is interesting. But it is important to note that even the minimum leakage fraction required by coexistence (0.055 for leucine and 0.095 for lysine) is far larger than the actual values we fit from experimental data (0.0066 for leucine and 0.011 for lysine), suggesting that the two engineered amino acid auxotrophies may not be able to coexist in chemostat at dilution rate equal to 0.1 h^{-1} (they may coexist at lower dilution rates). These discussions about Fig. 3D can be found in lines 253-261 of the revised manuscript.

Reply to Referee #2

Comment 1: *"In this manuscript, the Authors provide a detailed study of a consumer-resource model augmented by explicit cross-feeding dynamics of metabolites and resource dynamics. The Authors compare the model output to three experiments between cross-feeding E. coli strains at increasing levels of complexity, and discuss the results in the light of ecological interactions and community robustness. The Authors propose that by fitting the community dynamics to their modeling framework one can quantify exchange rates that are difficult to measure experimentally. I have enjoyed reading the manuscript, which I find interesting and well written. I agree with the Authors conclusion, but I have one major concern that I think should be addressed.*"

Author Reply: We are pleased that the referee enjoyed reading our paper and agreed with our conclusions.

Comment 2: "*In the introduction and in the discussion, the Authors mention the fact that manual fitting of a model has the downside of being subjective, time consuming and requiring an expert operator. I would add that perhaps the main downside of manual fitting is that one cannot infer correlations among parameters. Especially for chemical reaction models, where chemical species are produced and consumed at unknown rates, fitting parameters can be highly correlated because one can often rescale production and consumption rates to achieve the same average species concentrations. Especially given that the Authors propose this as a framework that can be used to quantify exchange rates that are hard to measure experimentally, I think that the paper requires a rigorous parameter identification approach. At least for the two-species communities, it should be feasible to adopt Markov-Chain-Monte-Carlo methods to estimate the posterior distribution of the parameters, and highlight possible correlations among them. This would also allow the Authors to report confidence intervals for various quantities that are inferred from the data.*"

Author Reply: We thank the referee for this constructive comment. We agree that a parameter sensitivity analysis is important to our paper. We followed the suggestion and ran Markov-Chain-Monte-Carlo (MCMC) simulations to estimate the posterior distribution of all free parameters (parameters whose values are not fixed by experimentally measure values) for the two 2-species

communities. We did try to run MCMC for the 14-species community model with >200 free parameters but it converged too slowly. The details of how we performed MCMC simulations, including brief description of the algorithm, prior distribution, and objective functions, are available in Materials and methods in the main text.

We now plot the posterior distributions of the free parameters and their pairwise correlations in Supplementary Fig. 3 (for unilateral cross-feeding community) and Supplementary Fig. 8 (for bilateral cross-feeding community). We also report the corresponding 25%, 50% and 75% percentiles as well as 95% confidence interval of their posterior distributions in Supplementary Table 1 (for unilateral cross-feeding community) and Supplementary Table 2 (for bilateral cross-feeding community). For all free parameters, their best-fit values through manual calibration are within the range of their posterior distributions. With the exception of a few cases, we did not find strong correlations among parameters, suggesting that these parameters have independent effects on the model behavior. We discuss the new results from the parameter sensitivity analysis in the main text (lines 151-167 for unilateral cross-feeding model and lines 233-247 for bilateral cross-feeding model). We also acknowledge downside of manual fitting in the inference of parameter correlations in the discussion (lines 497-502).

Comment 3: "*In line 128, the Authors mention that they used Monod kinetics for the cell death. My interpretation of this statement is that cell death is a saturating function of some chemical species or of cell density, but this doesn't seem to be the case looking at the equations, where cell death occurs at a constant rate.*"

Author Reply: We recognize that our explanation was incorrect. We rewrote the sentence (lines 129-130) to clarify that cell death rate was modeled not by Monod kinetics but by first-order kinetics with constant specific mortality rate.

Comment 4: "*Line 165-167: it would help to state explicitly here that the strains that are auxotroph for lysine and leucine can export them to the extracellular environment*"

Author Reply: We now explicitly mention that the two strains only export amino acids that their partners miss, and that this creates a bidirectional, obligate relationship (lines 217-218).

Comment 5: "*Supplementary Tables: I would suggest adding a column with literature values for those best-fit parameters that can be compared to experimentally-measured values*."

Author Reply: We thank the referee for the suggestion. We added a column "Literature" in each of Supplementary Table 1-3 and listed the literature values when available.

Bibliography

Barabás, G., D'Andrea, R., and Stump, S.M. (2018). Chesson's coexistence theory. Ecol. Monogr. *88*, 277–303.

Goldford, J.E., Lu, N., Bajić, D., Estrela, S., Tikhonov, M., Sanchez-Gorostiaga, A., Segrè, D., Mehta, P., and Sanchez, A. (2018). Emergent simplicity in microbial community assembly. Science *361*, 469–474.

Hoek, T.A., Axelrod, K., Biancalani, T., Yurtsev, E.A., Liu, J., and Gore, J. (2016). Resource Availability Modulates the Cooperative and Competitive Nature of a Microbial Cross-Feeding Mutualism. PLoS Biol. *14*, e1002540.

Liao, C. (2018). Integrated modeling of bacterial metabolism.

Liao, C., Blanchard, A.E., and Lu, T. (2017). An integrative circuit-host modelling framework for predicting synthetic gene network behaviours. Nat. Microbiol. *2*, 1658–1666.

Marsland, R., Cui, W., Goldford, J., Sanchez, A., Korolev, K., and Mehta, P. (2019). Available energy fluxes drive a transition in the diversity, stability, and functional structure of microbial communities. PLoS Comput. Biol. *15*, e1006793.

Mee, M.T., Collins, J.J., Church, G.M., and Wang, H.H. (2014). Syntrophic exchange in synthetic microbial communities. Proc. Natl. Acad. Sci. USA *111*, E2149-56.

Momeni, B., Xie, L., and Shou, W. (2017). Lotka-Volterra pairwise modeling fails to capture diverse pairwise microbial interactions. Elife *6*.

Rosenzweig, R.F., Sharp, R.R., Treves, D.S., and Adams, J. (1994). Microbial evolution in a simple unstructured environment: genetic differentiation in Escherichia coli. Genetics *137*, 903–917.

Rozen, D.E., and Lenski, R.E. (2000). Long-Term Experimental Evolution in Escherichia coli. VIII. Dynamics of a Balanced Polymorphism. Am. Nat. *155*, 24–35.

Taillefumier, T., Posfai, A., Meir, Y., and Wingreen, N.S. (2017). Microbial consortia at steady supply. Elife *6*.

Tilman, D. (1982). Resource competition and community structure. Monogr Popul Biol *17*, 1–296.
